# Evolution of plant senescence

**DOI:** 10.1186/1471-2148-9-163

**Published:** 2009-07-14

**Authors:** Howard Thomas, Lin Huang, Mike Young, Helen Ougham

**Affiliations:** 1 IBERS, Aberystwyth University, Ceredigion, SY23 3DA, UK; 2 IBERS, Aberystwyth University, Plas Gogerddan, Aberystwyth, Ceredigion, SY23 3EB, UK

## Abstract

**Background:**

Senescence is integral to the flowering plant life-cycle. Senescence-like processes occur also in non-angiosperm land plants, algae and photosynthetic prokaryotes. Increasing numbers of genes have been assigned functions in the regulation and execution of angiosperm senescence. At the same time there has been a large expansion in the number and taxonomic spread of plant sequences in the genome databases. The present paper uses these resources to make a study of the evolutionary origins of angiosperm senescence based on a survey of the distribution, across plant and microbial taxa, and expression of senescence-related genes.

**Results:**

Phylogeny analyses were carried out on protein sequences corresponding to genes with demonstrated functions in angiosperm senescence. They include proteins involved in chlorophyll catabolism and its control, homeoprotein transcription factors, metabolite transporters, enzymes and regulators of carotenoid metabolism and of anthocyanin biosynthesis. Evolutionary timelines for the origins and functions of particular genes were inferred from the taxonomic distribution of sequences homologous to those of angiosperm senescence-related proteins. Turnover of the light energy transduction apparatus is the most ancient element in the senescence syndrome. By contrast, the association of phenylpropanoid metabolism with senescence, and integration of senescence with development and adaptation mediated by transcription factors, are relatively recent innovations of land plants. An extended range of senescence-related genes of *Arabidopsis *was profiled for coexpression patterns and developmental relationships and revealed a clear carotenoid metabolism grouping, coordinated expression of genes for anthocyanin and flavonoid enzymes and regulators and a cluster pattern of genes for chlorophyll catabolism consistent with functional and evolutionary features of the pathway.

**Conclusion:**

The expression and phylogenetic characteristics of senescence-related genes allow a framework to be constructed of decisive events in the evolution of the senescence syndrome of modern land-plants. Combining phylogenetic, comparative sequence, gene expression and morphogenetic information leads to the conclusion that biochemical, cellular, integrative and adaptive systems were progressively added to the ancient primary core process of senescence as the evolving plant encountered new environmental and developmental contexts.

## Background

We present evidence that the genes and metabolism representing the core of the process of senescence in modern land-plants can be traced back to primaeval unicellular photoautotrophs and that specific elaborations and regulatory mechanisms have been progressively added to the senescence program at decisive points during plant evolution. Senescence is a prominent characteristic of angiosperm morphogenesis and ecology. The term is applied at all levels of plant biology, from processes at the biome-wide scale that define the season of autumn in temperate climates [1,2] through to the cellular dimension in the terminal development of individual tissues and organs [3,4]. Plant senescence is commonly considered to be programmed [5] and several genes have been identified as essential for normal initiation and/or execution of the syndrome [6,7]. Here we focus particularly on the metabolism of pigments in green tissues and the colorful lateral organs and structures of angiosperms [8,9] and, based on comparative cell biology and protein sequence relationships across the evolutionary range of plants and their phototrophic ancestors, we reconstruct a progression of genetic events leading to the establishment of senescence as an essential element in the terrestrial plant life-cycle.

### Evolutionary origin of photosynthetic pigments

A challenging question sometimes asked of biologists by children and physicists is: if leaves are solar panels, why are they green and not black [10-12]? This query in turn implies further questions about the nature of the earliest life-forms, how they used light as an energy-source, how the photosynthetic apparatus became organised and reorganised during evolution and (of particular relevance to the present paper) the evolutionary origin of pigment metabolism and its control in plant development. The light absorption profile of chlorophyll-based green membranes is characterised by a broad gap in the middle of the visible wavelength range. It has been proposed that the earliest phototrophs had light receptors that occupied the centre of the spectrum between the red and blue peaks of chlorophyll-based green membranes, and that chlorophyll evolved as a complementary pigment, filling in the missing wavelengths at the edges of the spectrum. According to this hypothesis the earliest photoautotrophs may have been similar to purple bacteria in using bacteriorhodopsin as the light-intercepting pigment-protein and bacteriochlorophyll at the reaction centre, driving a proton pump coupled to ATPase [10,13,14]. Phylogenetic analyses based on genes for photosynthesis as well as other markers such as rRNA and heat shock proteins are suggesting a number of alternative trajectories for the early evolution of pigment metabolism and organisation, but the widespread prevalence of horizontal gene transfer makes it difficult to agree the definitive scenario [13,15]. We can be certain, however, that isoprenoid-derived pigments continued to carry out the function of intercepting light in the middle of the visible spectrum while chlorophyll adopted an increasingly prominent role in light harvesting as well as charge separation, leading to modern streptophytes including land plants.

Phylogenetic analysis of genes for Mg-tetrapyrrole biosynthesis indicates that anoxygenic photosynthetic organisms are ancestral to oxygen-evolving cyanobacteria and that the pathway in purple bacteria may be closest to that of the most ancient phototrophs [16]. By contrast to chlorophyll synthesis, the catabolic side of chlorophyll metabolism has not hitherto been examined in molecular phylogenetic terms, though a detailed survey of the enzymological properties of RCC reductase (RCCR) in a range of terrestrial species carried out by Hörtensteiner et al. [17] allowed some inferences to be drawn about the evolution of the pigment breakdown pathway in land plants. In the present paper we analyse the occurrence of genes for two key enzymes of chlorophyll catabolism (phaeophorbide a oxygenase – PaO – and RCCR; [18]), one post-translational regulator (Sgr; [19]) and two catabolite transporters (WBC23 and MRP2; [20]) in the lineages leading to modern angiosperms.

### From photosynthetic eukaryotes to the invasion of land by plants

The significant early events in the evolution of the photosynthetic apparatus comprised diversification of reaction centre and antenna structure, based on chlorophyll and isoprenoids, and organisation and delineation of oxygenic and anoxygenic photosynthesis. According to Strother [21] 'the endosymbiotic origin of plastids...indicates that perhaps all of the original host cell lineages for the algae were phagotrophic...' Based on nutrient conditions and the evolution of food webs in the Palaeo- and Meso-proterozoic ocean [21,22], it is reasonable to suppose that facultative autotrophy would have been the rule in early photosynthetic organisms. The formation of endosymbiotic relationships resulting in the evolution of plastid-containing eukaryotic cells probably occurred just once in the lineage, although there is evidence of several secondary events, including horizontal gene transfer, too [23-25]. It seems likely that the foundation endosymbiont was mixotrophic in character and that the earliest green eukaryotes were facultatively autotrophic [21,26]. This is significant for the origin of plant senescence because aspects of the biochemistry and cell biology of plastids in terrestrial plants suggest that the transition from chloroplast to the plastids of senescing leaves (gerontoplasts) or fruit and floral parts (chromoplasts) is a change in status from auto- to hetero-trophy [27]. A strikingly senescence-like response during the change of trophic condition on removal of light and nitrogen source is seen in cultures of *Auxenochlorella *(formerly *Chlorella*) *protothecoides*. Cells turn yellow, accompanied by changes in protein and enzyme activities that resemble those of senescing mesophyll cells [28], and a red pigment is secreted into the medium [29-31]. Chemical analysis shows the pigment to be a bilin derived from chlorophyll by removal of phytol and Mg and by oxygenolysis of the methine bridge between pyrrole groups A and B [32]. This structure and its biochemical origin relate directly to the metabolic sequence for chlorophyll catabolism in angiosperms [33]. Similar production of chlorophyll catabolites has been reported in a *Chlamydomonas *mutant [34]. The luciferin of the bioluminescent dinoflagellate *Pyrocystis lunula *is also a bilin degradation product of chlorophyll, though in this case the chlorin macrocycle is opened between rings A and D [35]. A luciferin of similar structure is found in the krill organism *Euphausia pacifica *[35,36], presumably originating in ingested phytoplankton. We conclude that at least part of the enzymic pathway of chlorophyll catabolism was present during the aquatic phase of plant evolution and this has led us to seek phylogenetic continuity between genes for trophic responses and pigment catabolism from unicellular algae to angiosperms.

Amongst the genes expressed during the auto- to hetero-trophic transition in *Auxenochlorella *is an amino acid permease, dee4 [28]. Transcripts of the *Arabidopsis *homologue of dee4, At1g44100, were identified in isolated nuclei of *Arabidopsis *phloem cells, suggesting a role in nitrogen translocation from sources to sinks [37]. Bleecker [38] considered nutrient salvage to be a driving force in the evolution of the plant senescence program. Nutrient recovery implies structural differentiation into source and destination tissues, with a transport system between them [39,40]. To assess their relevance to the evolution of nutrient relations during senescence, we carried out protein sequence tree and expression analyses of dee4 and At1g44100 respectively. We also analysed a NAC gene with a regulatory function in organ development and senescence. Uauy et al. [41] isolated NAM-2, a NAC transcription factor, by positional cloning and demonstrated a regulatory role in nutrient redistribution from senescing leaves to developing grain in cereals. AtNAP, a NAM-like factor, expression of which is closely associated with the senescence process of *Arabidopsis *rosette leaves [42], was included in our analyses of expression and taxonomic distribution.

From endosymbiont origins of photosynthetic eukaryotes to angiosperms is a sequence of major events including differentiation of two- and three-dimensional body plans and emergence from the aquatic environment onto land [43]. Cell specialisation, intra- and inter-cellular signalling and elaboration of body plan are regulated by homeoprotein transcription factors and the integration of senescence into morphogenetic and adaptive programs is likely to be apparent in the phylogenetic record. Prominent amongst the regulators of signalling networks activated during plant senescence are transcription factors of the Wrky family. Sequence analysis of Wrky53, the most intensively studied of the senescence-related signal-transduction components [44], suggests the point during evolution at which such factors may have became available for networking with the core senescence program.

### Appearance of coloured organs in land-plants driven by coevolution

As well as promoting diversification of structure, and adaptation to terrestrial ecological niches, achieving landfall eventually exposed plants to new biotic and abiotic interactions. The ancient role of carotenoids as quenchers of excess light energy intercepted by photosystems [45] acquired new significance in environments where mismatch between photon flux, temperature and the availability of water and nutrients is the rule and a strongly oxidising atmosphere supports the uncontrolled propagation of damage by reactive chemical species. Coevolution between plant pigments and the visual systems of insects and other animals introduced a new role for carotenoids as signalling molecules, advertising to pollinators, to dispersers or to herbivores and other predators [46,47]. Investigations of the phylogenetic origin of carotenoids in relation to colour changes in senescence and ripening are frustrated by the ubiquity and antiquity of the corresponding genes and the inordinate degree of horizontal gene transfer apparent in the evolutionary record [48,49]. Nevertheless, we have identified four genes associated with the subcellular organisation and developmental regulation of carotenoid metabolism in senescing and ripening tissues that provide insights into how this aspect of senescence may have evolved. Lipid bodies (plastoglobules) accumulate during the transition from chloroplast to gerontoplast or chromoplast [50]. They have been observed in the plastids of *Auxenochlorella *during the switch from auto- to hetero-trophy [51]. Plastoglobule-like lipid bodies are very widely distributed amongst prokaryotes [52]. Existing and newly-synthesised carotenoids concentrated in plastoglobules are responsible for the colours of fruits and senescing leaves in some species [8,53,54]. We examined the distribution of sequences similar to PAP-Fibrillin, a plastoglobule protein with functions in plastid development and stress responses [55].

Carotenoid metabolism in senescence and ripening is closely integrated with the transdifferentiation of chloroplasts into gerontoplasts and chromoplasts. Chromoplast development is regulated by Or (dnaJ-type) chaperone/transcription factors [56], two of which we included in our analyses. Carotenoid derivatives also have hormonal and cell signalling roles in plants. Genes of the Carotenoid Cleavage Dioxygenase (CCD) group are known to participate in regulation of a number of aspects of development, including leaf senescence [57]. Accordingly, we carried out an analysis of the protein sequences related to that of senescence-associated CCD8.

Another major class of pigments in land-plants comprises products of phenylpropanoid metabolism [58]. Anthocyanins are considered to have adaptive value both in stress responses and in signalling to beneficial or predatory animals, and there is an extensive literature on the significance of the range of possible anthocyanin functions in senescing leaves [8,59]. By contrast with carotenoids, which are highly hydrophobic and confined to the lipophilic environment of plastid membranes and plastoglobules, anthocyanins are water-soluble and accumulate in the cell vacuole [60]. The behaviour of the two classes of pigment during senescence is indicative of the state of their respective subcellular compartments. The anthocyanin responsible for the red colour of autumn leaves is commonly cyanidin-3-glucoside. The final step in its synthesis is catalysed by anthocyanidin 3-O-glucosyltransferase, encoded by the Bronze1 (Bz1) gene [61]. A myb transcription factor C1 regulates expression of the anthocyanin pathway [62]. We included Bz1 and C1 in our evolutionary survey of senescence-associated genes. As a senescence-related marker for the evolution of specific tonoplast membrane functions we also selected MRP2, a chlorophyll catabolite transporter [63]. The proteome of the vegetative plant cell vacuole is consistent with a lytic role in defence and tissue differentiation [64]. Transposon insertional mutagenesis of See2, a gene of maize encoding a vacuolar processing endopeptidase (VPE) has a range of effects on plant development, including delay of senescence [65]. We carried out an evolutionary survey of See2 and confirmed senescence-related expression of the *Arabidopsis *homologue At4g32940.

### Relationships between the ontogeny and phylogeny of plant senescence

As well as analysing the distribution across plant and microbial taxa of the selection of senescence-related protein sequences described above, we applied the clustering tools in Genevestigator to an extended group of *Arabidopsis *genes with likely functions in senescence, with the object of identifying common regulatory units. It is also of interest to seek evidence for homologies between terminal physiological changes in different organs, which in turn would begin to answer the question as to whether there is a common senescence program which operates, with variations, in all plant cells, tissues and organs.

## Results

Gene expression profiles, protein structures and molecular phylogenies are discussed in relation to chlorophyll catabolism and its control, homeoprotein transcription factors with known functions in senescence, metabolite transporters, carotenoid metabolism and anthocyanin biosynthesis (Table 1). Many senescence-associated genes have been described but the physiological functions of relatively few of them have been established, and fewer still have been demonstrated to be necessary for initiation or execution of the syndrome [6,7,9]. Those listed in Table 1 have been shown, by mutation, transgenic manipulation or biochemical analysis, to have defined roles in senescence. The tools of phylogenetic analysis were applied to amino acid rather than nucleotide sequences to avoid difficulties with codon usage across the very wide taxonomic scale considered in this study, though where a particular taxon yielded no hit when a critical protein was BLASTed, we checked for the existence of corresponding pseudogenes or other untranslated nucleotide sequence. Since we are principally interested in the first detectable occurrence and subsequent evolutionary continuity of particular protein sequences, we have not explicitly distinguished between orthologues and paralogues, a known issue when establishing phylogenetic and functional relationships [66]. The relative paucity of sequence data for many of the species examined is an acknowledged limitation in this study but, with a few exceptions discussed in detail, the analyses reported here make a consistent and biologically plausible picture.

**Table 1:** Proteins and their corresponding *Arabidopsis *genes with known functions in senescence, selected for phylogenetic and expression analyses.

		**Protein** **(UniProt code)**	***Arabidopsis *gene (AGI code)**	**Reference**
	1	*Arabidopsis *RCCR(NP_195417)	At4g37000	[68,132]
	
**Chlorophyll catabolism**	2	*Arabidopsis *PaO (NP_190074)	At3g44880	[18,31]
	
	3	*Arabidopsis *Sgr1 (NP_567673)	At4g22920	[19,69,70]
	
	4	*Arabidopsis *WBC23 – envelope FCC transporter (NP_850781)	At5g06530	[20]

	5	*Arabidopsis *Wrky53 (NP_194112)	At4g23810	[41,149]
	
**Cell and tissue specialisation**	6	*Arabidopsis *AtNAP (NP_564966)	At1g69490	[42]
	
	7	*Auxenochlorella *dee4 – amino acid permease (Q9XFY8)	At1g44100	[28]

	8	*Arabidopsis *Fibrillin (NP_192311)	At4g04020	[55,159]
	
**Plastid trans-differentiation**	9	*Arabidopsis *Carotenoid Cleavage Dioxygenase8 (NP_195007)	At4g32810	[57]
	
	10	*Brassica *dnaJ chaperone OrI (ABH07405)	At5g61670	[56,80]
	
	11	*Arabidopsis *dnaJ chaperone OrII (NP_851031)	At5g06130	[56]

	12	*Zea *Bronze1 (P16167)	At5g17050	[61]
	
**Vacuole function**	13	*Zea *myb C1 anthocyanin TF (P10290)	At4g34990	[62]
	
	14	*Arabidopsis *AtMRP2 ATP transporter (NP_181013)	At2g34660	[63,139]
	
	15	*Zea *See2 (CAB64545)	At4g32940	[64,82]

**Figure 1:**
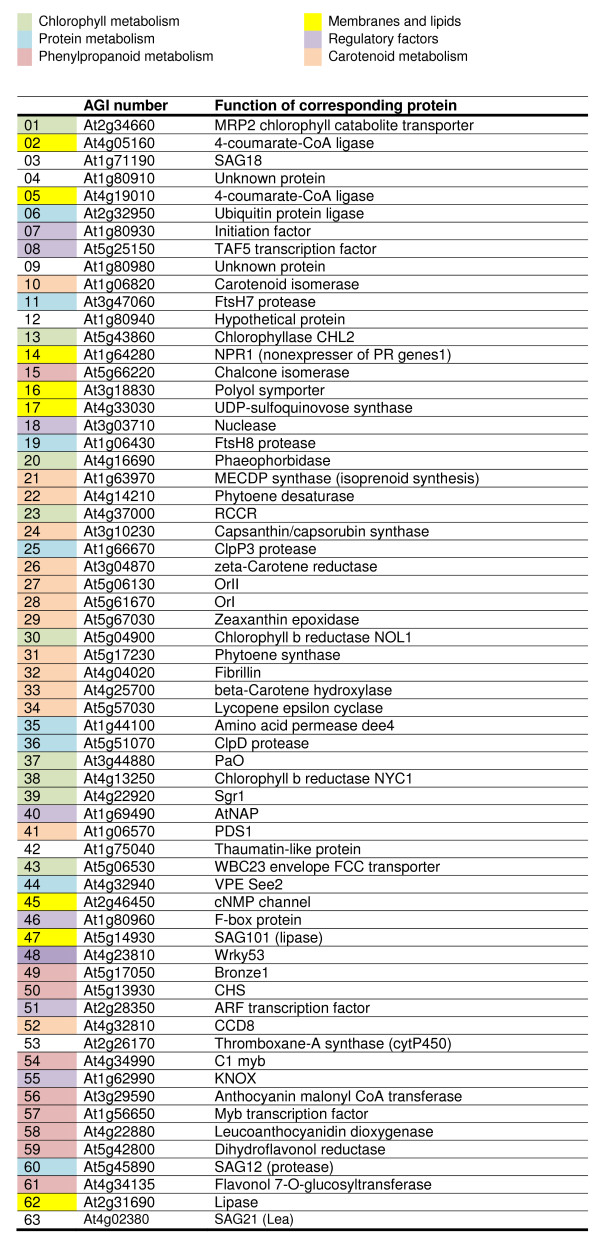
***Arabidopsis *genes with functions and expression patterns related to senescence**. The list includes the genes in Table 1 plus senescence-enhanced genes identified by a search of the TAIR database. Genes are colour-coded according to broad functional categories, where known or inferred.

In most cases the trees are presented in unrooted radial form and are based on global alignments of protein sequences from one or two representative genera of each major taxon. Tree topologies for individual proteins often deviated from those predicted by the evolutionary relationships between the corresponding taxa. The non-congruence of gene and species trees is a well-described feature of molecular phylogenies and indeed is statistically to be expected [67]. Bootstrap values and branch lengths with their error estimations are presented in Additional Files 1 and 2 respectively. The range of senescence-related sequences (Table 1) is subsequently extended (Figure 1) for cluster analysis to reveal units of coexpression and developmental relationships amongst plant structures with respect to their senescence programs.

### Chlorophyll catabolism

**Figure 2:**
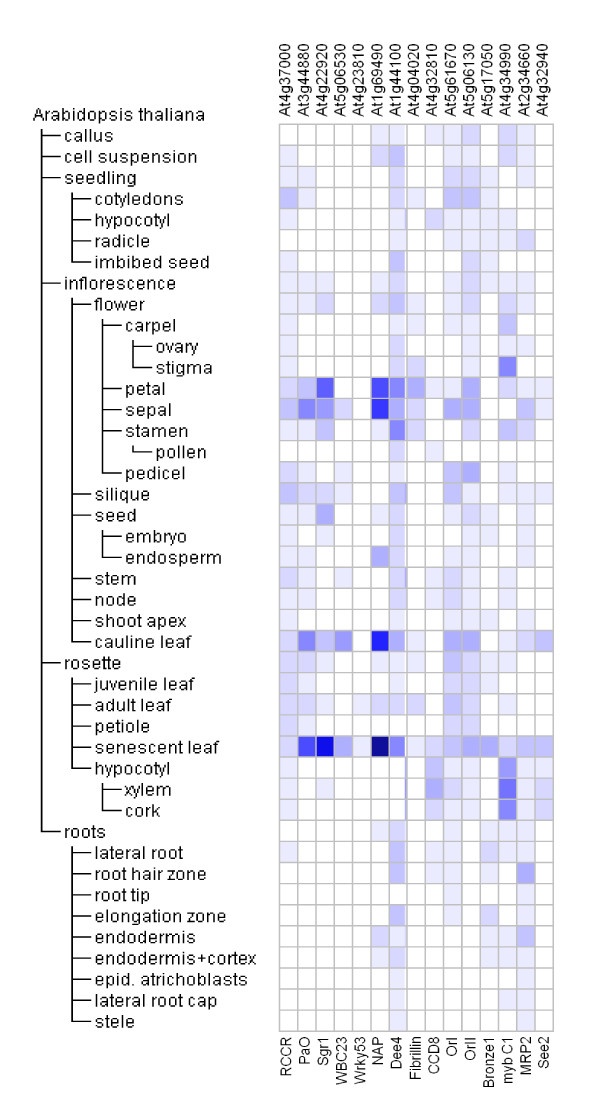
**Genevestigator Anatomy expression profiles of 15 senescence-related *Arabidopsis *genes (L to R: RCCR, PaO, Sgr1, WBC23, Wrky53, NAP, dee4, Fibrillin, CCD8, OrI, OrII, Bronze1, myb C1, MRP2, See2)**. Heat maps represent expression level in Affimetrix^® ^arrays as colour intensity computed in Genevestigator according to the MAS5 normalization algorithm [192].

The observation of bilin products of chlorophyll catabolism in unicellular algae focuses attention on the enzymes responsible for opening the chlorin macrocycle, namely PaO and RCCR. Figure 2 is a Genevestigator heat map representation of the relative expression of all the *Arabidopsis *genes whose protein trees are reported in the present paper, including RCCR and PaO (AGI accession numbers respectively At4g37000 and At3g44880). Expression of RCCR is not particularly strong in senescing leaves. It gives a noticeably weak signal in roots and other non-green structures like pollen. PaO is also largely undetectable in non-green tissues but exhibits clear differential expression during development, being most prominent in senescing leaf, cauline leaf and sepal.

**Figure 3:**
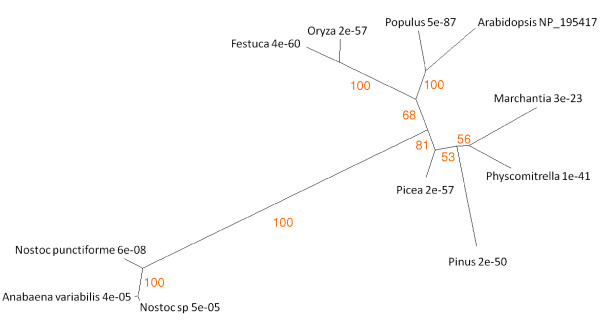
**Unrooted radial bootstrapped protein sequence tree for RCCR based on PHYLIP analyses using *Arabidopsis *RCCR (UniProt NP_195417) as the reference sequence**. Representative genera are shown, together with E values, indicating the numerical expectation that the alignment with the reference sequence has arisen by chance. Support values (in red) were determined by bootstrap analysis (Additional file 1a). Branch lengths are proportional to number of amino acid substitutions per position. Additional file 2a gives branch length values and confidence limits from the PROML outfile.

Sequence similarities to the protein corresponding to RCCR gene At4g37000 (UniProt accession number for the barley sequence Q8LDU4; [68]) were analysed. Figure 3 shows the relationships between these proteins from representative genera in tree form. Three major groupings are apparent. Angiosperms form one clade, with monocots and dicots resolved within it. Non-angiosperm land plants are grouped together. Cyanobacteria form a third clade, characterised by relatively high E scores, although examination of the sequences for conserved domains identified these cyanobacterial proteins as members of the RCCR family. For example SMART analysis recognised the RCCR conserved domain in the *Anabaena *protein, recording an extremely low E score of 4.80e^-159^. Protein and DNA BLAST searches, both of general databases and of the JGI *Chlamydomonas reinhardtii *site, failed to record a single hit within the green algae. The significance of the discontinuity in the distribution of RCCR across phyla is considered in the Discussion section.

**Figure 4:**
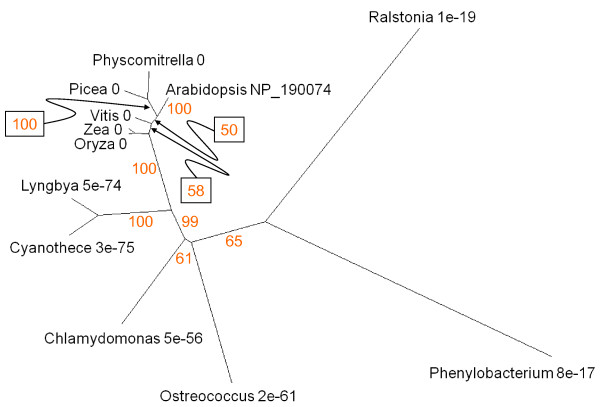
**Unrooted radial bootstrapped protein sequence tree for PaO based on PHYLIP analyses using *Arabidopsis *RCCR (UniProt NP_190074) as the reference sequence**. Representative genera are shown, together with E values, indicating the numerical expectation that the alignment with the reference sequence has arisen by chance. Support values (in red) were determined by bootstrap analysis (Additional file 1b). Branch lengths are proportional to number of amino acid substitutions per position. Additional file 2b gives branch length values and confidence limits from the PROML outfile.

**Figure 5:**
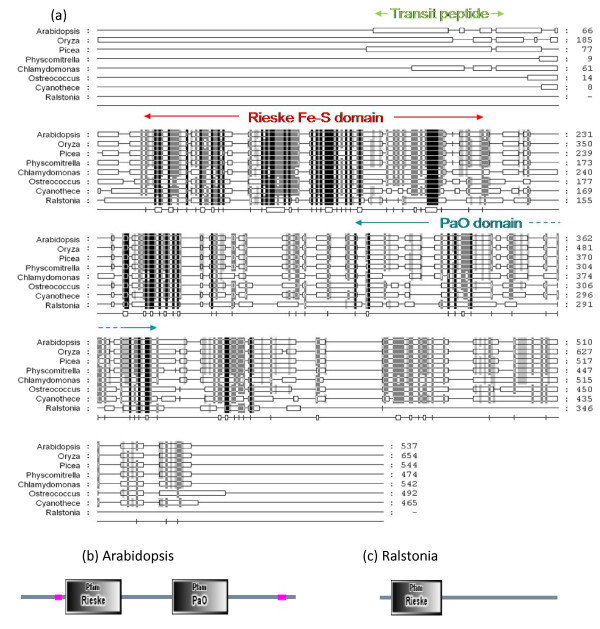
**(a) Comparison of PaO structures in representative genera by BLASTP alignment**. Shading density indicates % sequence similarity: black = 100%; dark grey = 80%; light grey = 60%. Conserved motifs are shown. Both the Rieske and PaO domains are present in the *Arabidopsis *sequence (b) and other species except that of the b-proteobacterium *Ralstonia *(c), in which the PaO motif is missing.

The tree representation of proteins similar to the *Arabidopsis *PaO gene At3g44880 (UniProt accession number NP_190074; Figure 4) reveals that the land plants form a group of closely related sequences (E value of 0). The cyanobacteria, represented by *Cyanothece *and *Lyngbya*, make a distinct cluster, the green algae *Chlamydomonas *and *Ostreococcus *make another and the proteobacteria *Phenylobacterium *and *Ralstonia *are more remotely related. The protein sequences of eukaryotes and cyanobacterial species include conserved Rieske Fe-S and PaO motifs (Figure 5). The *Ralstonia *sequence has a Rieske Fe-S domain near the N terminus but no PaO motif (Figure 5c). This is generally true of bacterial sequences with similarity to PaO. The *Ralstonia *protein is recognised as a member of the HcaE phenylpropionate dioxygenase multidomain group, which may be close to the evolutionary source of the PaO gene of phototrophs. It is striking that the cyanobacterial branch has a higher support value and their sequences have a higher similarity to those of land plants than do those of the eukaryotic green algae (Figure 4).

**Figure 6:**
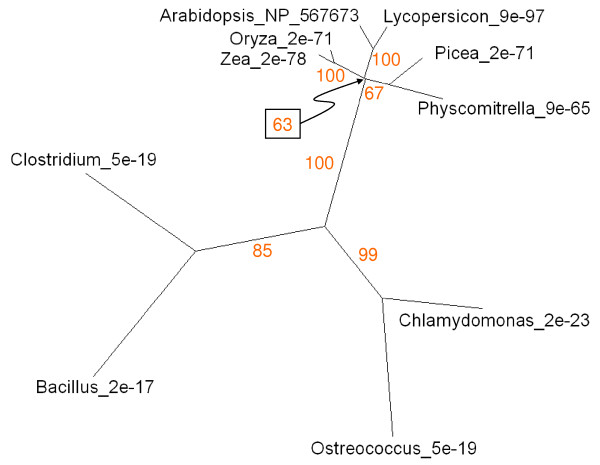
**Unrooted radial bootstrapped tree for plant and bacterial Sgr-like proteins based on PHYLIP analyses using *Arabidopsis *Sgr1 (UniProt NP_567673) as the reference sequence**. Representative genera are shown, together with E values, indicating the numerical expectation that the alignment with the reference sequence has arisen by chance. Support values (in red) were determined by bootstrap analysis (Additional file 1c). Branch lengths are proportional to number of amino acid substitutions per position. Additional file 2c gives branch length values and confidence limits from the PROML outfile.

**Figure 7:**
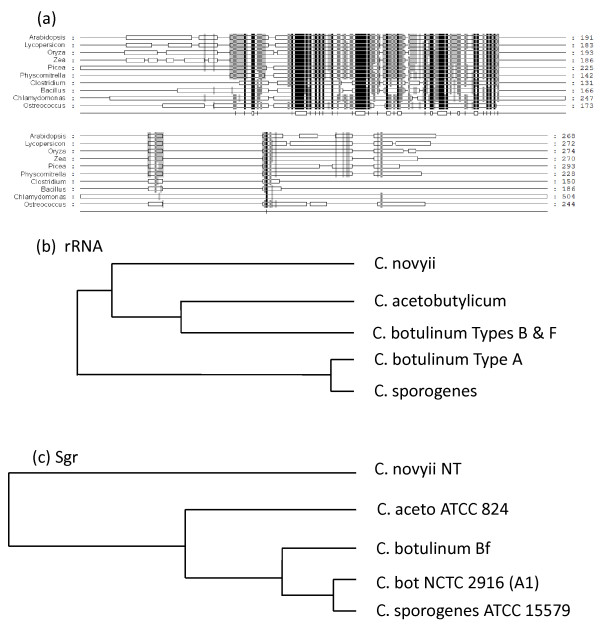
**Structures and phylogenies of *Clostridium *Sgr proteins**. (a) Comparison of Sgr structures in representative plant and Firmicute genera by BLASTP alignment. Shading density indicates % sequence similarity: black = 100%; dark grey = 80%; light grey = 60%. (b) Unrooted tree of phylogenetic relationships between *Clostridium *spp based on sequence alignments of ribosomal RNA [71,72]. (c) Protein tree of Sgr-like sequences from clostridia.

Stay-green (Sgr) is a post-translational regulator of chlorophyll-protein complex degradation [19,69,70]. The Genevestigator profile shows a tightly-specified expression pattern, with highest transcript abundance in senescent leaf and significant signal in floral parts (Figure 2). The sequence of the protein corresponding to *Arabidopsis *Sgr1 gene At4g22920 (UniProt accession number NP_567673) was aligned with similar proteins from other species and the results were used to generate the tree shown in Figure 6. Dicots, monocots and non-flowering land plants form three distinct groupings, with low E values that gradually increase with evolutionary distance from the reference species. Between the land plant and green algal clades there is a marked step up in E value. Grouped with the algae is a well-supported outlying clade, comprising a number of firmicutes (clostridia and bacilli). We further analysed the features of the Sgr-like genes in these species to determine whether Sgr may be indicative of a photosynthetic *Clostridium *ancestor, possibly something like the firmicute *Heliobacterium*. The rRNA [71,72] and Sgr trees are similar (Figure 7), but not congruent, and only 14 out of a total of 283 genomes searched (BLASTP at the JGI IMG site) contained genes encoding Sgr homologues. These were restricted to the genera *Clostridium *(11 of 44 genomes) and *Bacillus *(3 of 41 genomes) and there was no obvious conservation of genome context of Sgr between these organisms. We therefore conclude that Sgr is either an ancestral feature, which has been lost in all but a few remaining lineages; or, more likely, a feature that has been acquired more than once via horizontal gene transfer (Figure 7).

**Figure 8:**
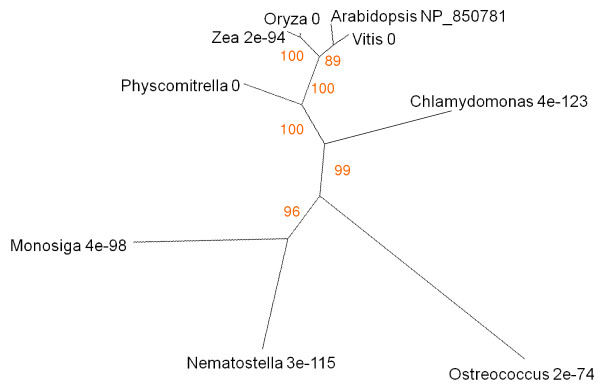
**Unrooted radial bootstrapped protein sequence tree for family of ATP-dependent bilin transporters based on PHYLIP analyses using *Arabidopsis *WBC23 (UniProt NP_850781) as the reference sequence**. Representative genera are shown, together with E values, indicating the numerical expectation that the alignment with the reference sequence has arisen by chance. Support values (in red) were determined by bootstrap analysis (Additional file 1d). Branch lengths are proportional to number of amino acid substitutions per position. Additional file 2d gives branch length values and confidence limits from the PROML outfile.

Aubry [20] identified WBC23, a transporter in the plastid envelope responsible for removing the linear tetrapyrrole product of the PaO-RCCR reaction. The corresponding gene, At5g06530, is strongly expressed in senescing leaves (Figure 2). ATP-dependent membrane transporters are very widely distributed across all eukaryotes [73], as evidenced by hits, with low E values, on the cnidarian *Nematostella *and the choanoflagellate *Monosiga *when BLASTed against *Arabidopsis *WBC23 (UniProt code NP_850781; Figure 8). Land plants group together, with clear discrimination into monocots, dicots and mosses. *Chlamydomonas *and *Ostreococcus *are associated at some distance from the land plants and each other. At higher E values, but still significantly less than chance, BLAST also identifies alignments with proteins from representatives of various insect groups.

### Cell and tissue specialisation

**Figure 9:**
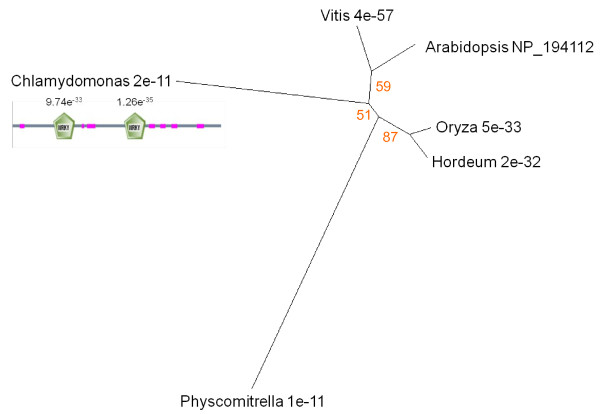
**Unrooted radial bootstrapped protein sequence tree for Wrky53 based on PHYLIP analyses using *Arabidopsis *Wrky53 (UniProt NP_194112) as the reference sequence**. Representative genera are shown, together with E values, indicating the numerical expectation that the alignment with the reference sequence has arisen by chance. Support values (in red) were determined by bootstrap analysis (Additional file 1e). Branch lengths are proportional to number of amino acid substitutions per position. Additional file 2e gives branch length values and confidence limits from the PROML outfile. Conserved domains in *Chlamydomonas *Wrky53-like protein are shown (low-complexity sequence indicated in pink).

In a study of genes expressed during *Arabidopsis *leaf senescence, Hinderhofer and Zentgraf [44] identified Wrky53 as a transcription factor with a major coordinating role in senescence regulation. Phylogenetic analysis of the Wrky family by Zhang and Wang [74] established that Wrky53 is a member of Group 3 that appeared before divergence of monocots and dicots but later than diversification of the bryophytes 160 – 330 million years ago (mya). Wrky53 is expressed exclusively in mature and senescing leaves (Figure 2), suggesting a role beginning in the earliest phase of senescence. Sequence similarities to the protein corresponding to Wrky53 gene At4g23810 (UniProt accession number NP_194112) were analysed and the resulting tree topology was in agreement with that of Zhang and Wang [74], with the exception of the appearance of a single hit in *Chlamydomonas *(Figure 9). The E score for this protein is 2e^-11 ^and analysis of conserved domains reveals two Wrky superfamily sequences with E-values of 9.7e^-33 ^and 1.26e^-35 ^respectively (Figure 9). We conclude that this represents a true algal Wrky53 homologue and is evidence that the appearance of Group 3 Wrkys may have been significantly earlier than the 330 mya originally inferred.

**Figure 10:**
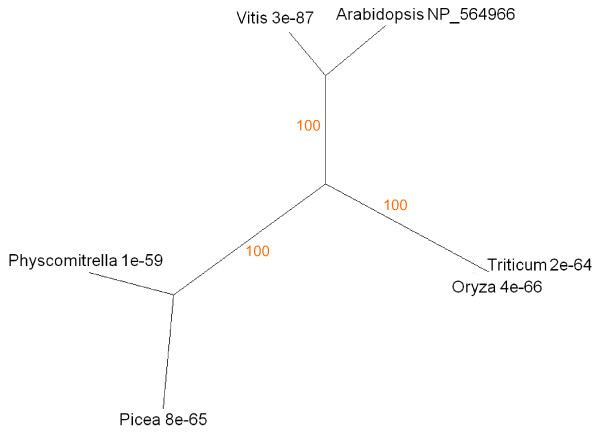
**Unrooted radial bootstrapped tree for NAP-like proteins based on PHYLIP analyses using *Arabidopsis *AtNAP (UniProt NP_564966) as the reference sequence**. Representative genera are shown, together with E values, indicating the numerical expectation that the alignment with the reference sequence has arisen by chance. Support values (in red) were determined by bootstrap analysis (Additional file 1f). Branch lengths are proportional to number of amino acid substitutions per position. Additional file 2f gives branch length values and confidence limits from the PROML outfile.

During senescence metabolites are relocated within the cell, from tissue to tissue and from source to sink organs. Uauy et al. [41] isolated a NAC transcription factor that corresponded to a major QTL for nutrient mobilisation from vegetative tissues during cereal grain-fill. Guo and Gan [42] also identified a senescence-associated NAC factor (AtNAP, AGI At1g69490) in *Arabidopsis*. Figure 2 reiterates the expression patterns reported by Guo and Gan [42], confirming strong upregulation in senescing leaves and high levels in cauline leaf, sepal and petal. Alignment of the AtNAP protein (UniProt NP_564966) showed similarity to the cereal sequence. The tree based on protein sequence alignments is presented in Figure 10. Exhaustive protein and nucleotide BLAST searches support the conclusion that NAP- and NAM-like proteins are exclusively found in land plants.

**Figure 11:**
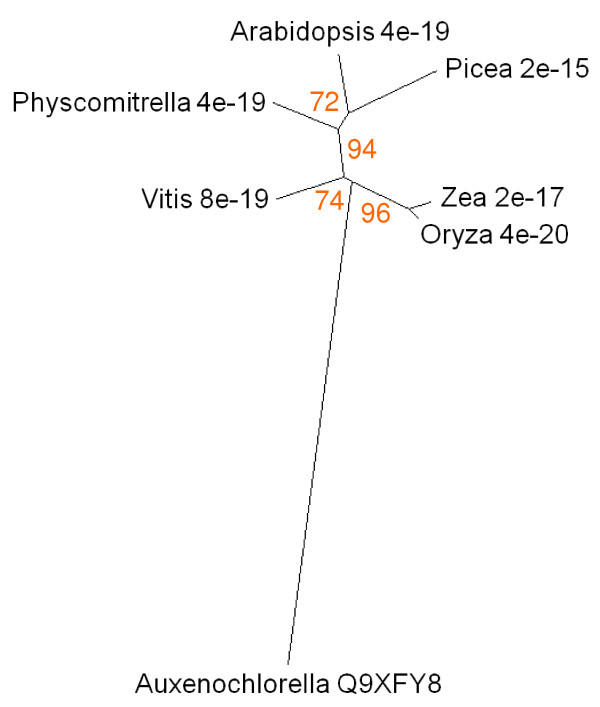
**Unrooted radial bootstrapped protein sequence tree for senescence-related amino acid permeases based on PHYLIP analyses using *Auxenochlorella *dee4 (UniProt Q9XFY8) as the reference sequence**. Representative genera are shown, together with E values, indicating the numerical expectation that the alignment with the reference sequence has arisen by chance. Support values (in red) were determined by bootstrap analysis (Additional file 1g). Branch lengths are proportional to number of amino acid substitutions per position. Additional file 2g gives branch length values and confidence limits from the PROML outfile.

A gene encoding an amino acid permease (dee4, UniProt Q9XFY8) is upregulated when *Auxenochlorella protothecoides *is transferred from auto- to hetero-trophic culture conditions; the *Arabidopsis *homologue (At1g44100) is also upregulated in senescing leaves (Figure 2) and has been identified in phloem [37]. Similar proteins to dee4 occur in angiosperms, mosses and conifers but are not present in taxa that diverged before the appearance of green algae (Figure 11).

### Gerontoplasts and chromoplasts

**Figure 12:**
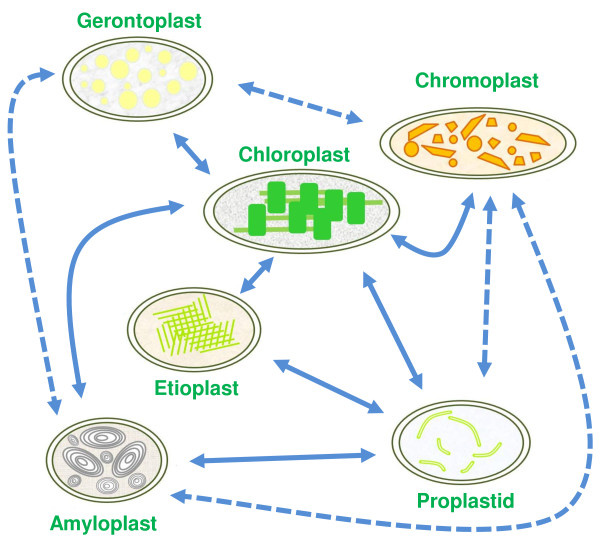
**The plastid development network (based on **[75,78]).

**Figure 13:**
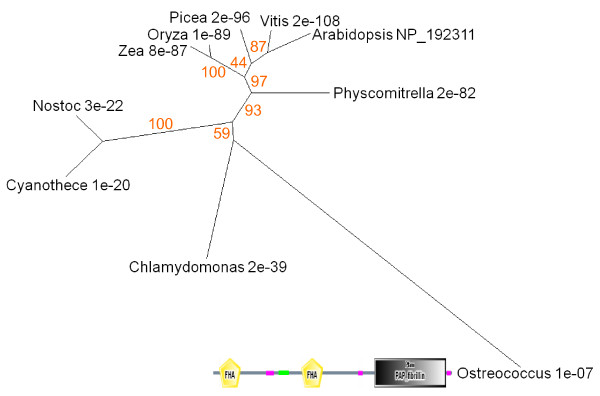
**Unrooted radial bootstrapped tree for Fibrillin-like proteins based on PHYLIP analyses using *Arabidopsis *PAP-Fibrillin (UniProt NP_192311) as the reference sequence**. Representative genera are shown, together with E values, indicating the numerical expectation that the alignment with the reference sequence has arisen by chance. Support values (in red) were determined by bootstrap analysis (Additional file 1h). Branch lengths are proportional to number of amino acid substitutions per position. Additional file 2h gives branch length values and confidence limits from the PROML outfile. The schematic diagram shows conserved domains within the *Ostreococcus *protein, including FHA (forkhead-associated) motifs, a coiled-coil sequence (green) and regions of low complexity (pink).

Plastids differentiate, dedifferentiate and morph from one type to another in a network of developmental transitions (Figure 12; [75-78]). Orange and red carotenoids are unmasked, concentrated or synthesised de novo in gerontoplasts and chromoplasts during senescence and ripening [8] and are sequestered in plastoglobules that become abundant as chloroplasts transdifferentiate [55,79]. We examined the expression and sequence relationships of *Arabidopsis *PAP-Fibrillin (UniProt NP_192311, AGI At4g04020), a plastoglobule-associated protein [55]. Genevestigator profiling shows expression to be absent from roots and to be prominent in cotyledons, floral parts and leaves (Figure 2). The protein tree for Fibrillin (Figure 13) reveals dicot and monocot clusters and a conifer branch with a comparatively low bootstrap value. Eukaryotic unicellular green algae and cyanobacteria make a distinct separate group. The *Ostreococcus *sequence with a high E score appears to be a chimeric protein comprising two additional FHA (forkhead-associated, putative nuclear-signalling) domains, separated from each other and the Fibrillin domain by regions of low complexity (Figure 13).

**Figure 14:**
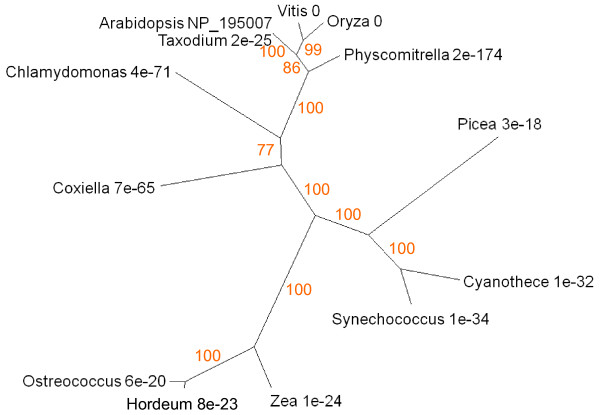
**Unrooted radial bootstrapped protein sequence tree for CCD8 based on PHYLIP analyses using *Arabidopsis *CCD8 (UniProt NP_195007) as the reference sequence**. Representative genera are shown, together with E values, indicating the numerical expectation that the alignment with the reference sequence has arisen by chance. Support values (in red) were determined by bootstrap analysis (Additional file 1i). Branch lengths are proportional to number of amino acid substitutions per position. Additional file 2i gives branch length values and confidence limits from the PROML outfile.

The gene Carotenoid Cleavage Dioxygenase8 (CCD8; UniProt NP_195007, AGI At4g32810) has a regulatory function in branch production, root growth, flower development and leaf senescence [57]. It is mostly expressed in shoots, particularly hypocotyl tissues, stem, node and senescent leaf (Figure 2). The protein is highly conserved from proteobacteria (eg *Coxiella*) to angiosperms (Figure 14) and a search for conserved domains establishes that CCD8 and its homologues are members of the RPE65 retinal pigment epithelial membrane protein superfamily which includes neoxanthin cleavage enzymes in plants and lignostilbene-α, β-dioxygenase in bacteria.

**Figure 15:**
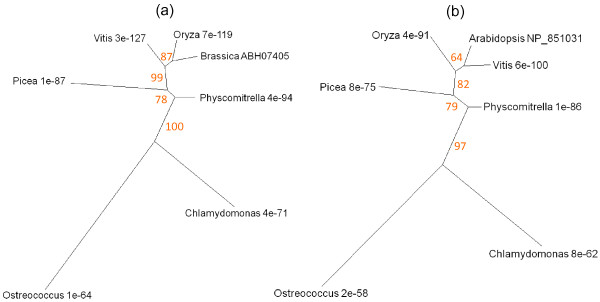
**Unrooted radial bootstrapped trees for two dnaJ chaperone-like proteins based on PHYLIP analyses using (a) OrI of *Brassica *(UniProt ABH07405) and (b) *Arabidopsis *OrII (UniProt NP_851031) as the reference sequences**. Representative genera are shown, together with E values, indicating the numerical expectation that the alignment with the reference sequence has arisen by chance. Support values (in red) were determined by bootstrap analysis (Additional file 1j and 1k). Branch lengths are proportional to number of amino acid substitutions per position. Additional file 2j and 2k give branch length values and confidence limits from the PROML outfiles.

The Or genes of *Brassica *encode proteins of the dnaJ chaperone class that play an important role in the differentiation of chromoplasts [56,80]. At5g61670 is the *Arabidopsis *gene corresponding to the cauliflower Or protein ABH07405 (UniProt), and is referred to here as OrI. It is expressed in floral parts, cotyledons and in cauline and senescent leaves (Figure 2). At5g06130 and NP_851031 are the AGI and UniProt codes for a second *Arabidopsis *Or-like dnaJ gene and protein respectively, designated OrII. The expression profile of this gene is broadly similar to that of At5g61670 (Figure 2), with somewhat stronger expression in senescing leaf, petal, stamen and pedicel. Trees of sequence relationships for OrI and OrII are presented in Figure 15. Both proteins are highly conserved from prasinophyceans to angiosperms. Prokaryotes gave no significant BLAST hit. The occurrence of Or-like genes in plants that do not differentiate chromoplasts, and their upregulation in a range of tissues including floral parts and senescing leaves (Figure 2), suggest that they may have a more generic function in plastid transdifferentiation.

### Vacuoles

**Figure 16:**
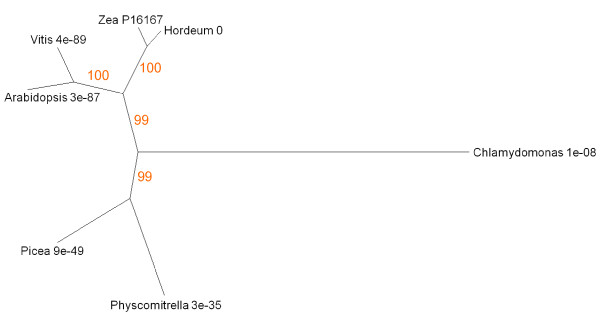
**Unrooted radial bootstrapped protein sequence tree for a family of anthocyanidin 3-O-glucosyltransferases based on PHYLIP analyses using *Zea *Bronze 1 (UniProt P16167) as the reference sequence**. Representative genera are shown, together with E values, indicating the numerical expectation that the alignment with the reference sequence has arisen by chance. Support values (in red) were determined by bootstrap analysis (Additional file 1l). Branch lengths are proportional to number of amino acid substitutions per position. Additional file 2l gives branch length values and confidence limits from the PROML outfile.

**Figure 17:**
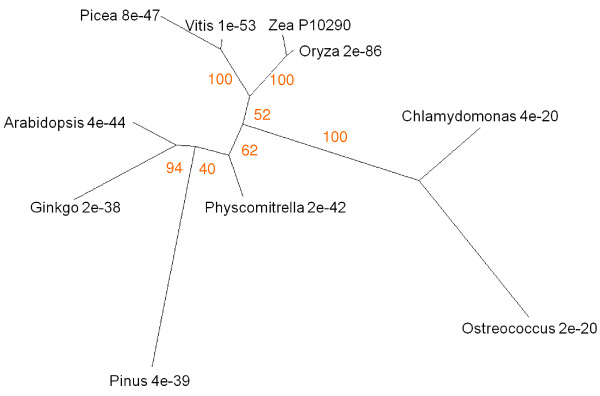
**Unrooted radial bootstrapped tree for plant and bacterial myb C1-like proteins based on PHYLIP analyses using *Zea *C1 transcription factor (UniProt P10290) as the reference sequence**. Representative genera are shown, together with E values, indicating the numerical expectation that the alignment with the reference sequence has arisen by chance. Support values (in red) were determined by bootstrap analysis (Additional file 1m). Branch lengths are proportional to number of amino acid substitutions per position. Additional file 2m gives branch length values and confidence limits from the PROML outfile.

Anthocyanin production and leaf yellowing are associated with senescence responses to nitrogen limitation, but are genetically independent [81]. We analysed the expression and protein sequence relationships of two genes with functions in anthocyanin biosynthesis. Bronze1 (Bz1) encodes the glycosyl transferase that synthesises the red vacuolar pigment cyanidin-3-glucoside. *Zea *Bz1 (UniProt P16167) was aligned with similar sequences; the homologous *Arabidopsis *gene is AGI At5g17050. The anthocyanin pathway is under the control of myb transcription factor C1 (UniProt accession number of *Zea *C1 P10290; AGI code of the homologous *Arabidopsis *gene At4g34990). Both genes are widely expressed in different organs and tissues (Figure 2). Bz1 is most prominent in senescing leaf. C1 is also expressed in senescing leaf but is most strongly represented in stigma and hypocotyl tissues. The Bz1 tree reveals the occurrence of homologues in angiosperms, conifers and mosses (Figure 16). At an E value of 1e^-08^, only a single BLAST hit was recorded for a non-tracheophyte. The *Chlamydomonas *protein has a UDP glycosyl transferase region towards the C terminal end but low similarity to Bz1 elsewhere in the polypeptide. A BLAST search against the *Chlamydomonas *protein identifies other plant UDP glycosyl transferases, but E scores are high, and the algal protein aligns more closely with a range of nucleoside glycosyl transferases from metazoa. We conclude that this algal protein has a distant structural relationship to tracheophyte Bz1-like sequences but functions as a transferase in a different metabolic pathway from that leading to anthocyanin synthesis. It follows that the anthocyanin pathway appeared relatively late in the evolution of senescence processes, around the time of cryptogam diversification. By contrast, genes related to C1, the myb transcription factor responsible for regulating expression of anthocyanin synthesis, are well represented throughout plant taxa, from *Zea *to *Ostreococcus *(Figure 17). BLAST also identifies similar genes in the actinomycete *Frankia *and other bacteria.

**Figure 18:**
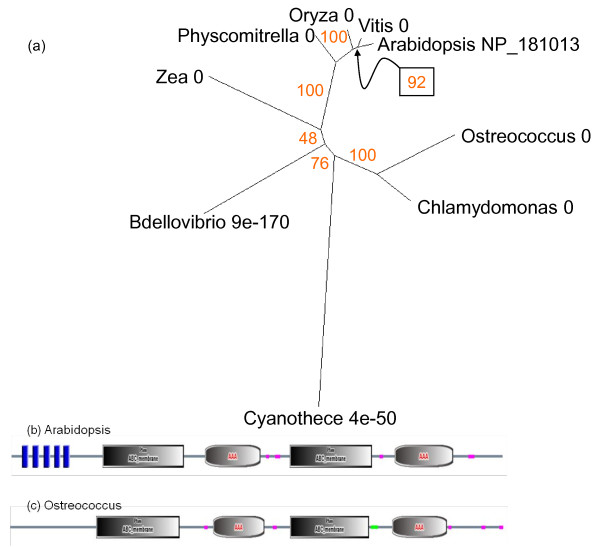
**(a) Unrooted radial bootstrapped protein sequence tree for ABC-type transporters based on PHYLIP analyses using to *Arabidopsis *AtMRP2 (UniProt NP_181013) as the reference sequence**. Representative genera are shown, together with E values, indicating the numerical expectation that the alignment with the reference sequence has arisen by chance. Support values (in red) were determined by bootstrap analysis (Additional file 1n). Branch lengths are proportional to number of amino acid substitutions per position. Additional file 2n gives branch length values and confidence limits from the PROML outfile. Conserved domains in (b) *Arabidopsis *and (c) *Ostreococcus *MRP2 sequences. AAA, ATPase region; blue block, transmembrane segment; pink block, low complexity region.

The final products of chlorophyll catabolism are deposited in the vacuole. They are moved across the tonoplast membrane by ATP mediated transporters of the MRP type [63]. MRP2 (AGI At2g34660) is expressed in senescent and cauline leaves as well as sepal, stamen and root tissues (Figure 2). Multidrug-resistance transporter proteins like AtMRP2 are very widely distributed across plants and animals. The tree of the corresponding protein (UniProt NP_181013) identifies a group of closely-related structures within angiosperms, mosses and green algae. Interestingly the MRP2-like protein of the predatory bacterium *Bdellovibrio *aligns to *Arabidopsis *with a lower E score than the cyanobacterial homologue (Figure 18a). BLAST also identifies a more distantly-diverged, but still highly similar, branch consisting of protozoa (Placozoa, Choanocyte) and vertebrates. The algal and angiosperm MRP2-like sequences have similar domain structures (Figure 18b, c).

**Figure 19:**
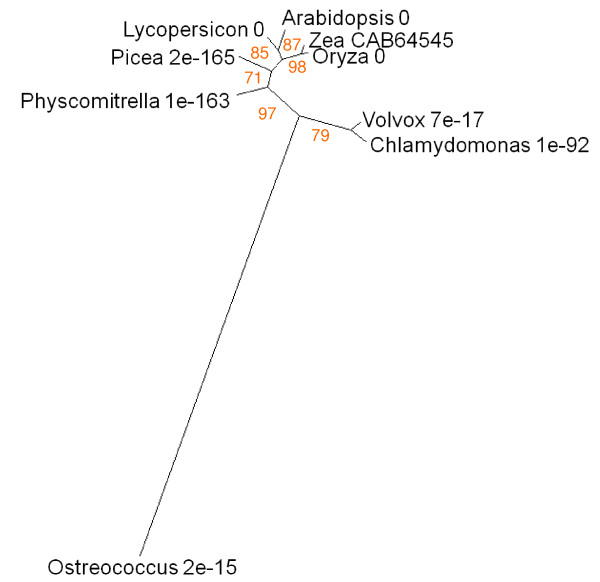
**Unrooted radial bootstrapped tree for VPE-like proteins based on PHYLIP analyses using *Zea *See2 (UniProt CAB64545) as the reference sequence**. Representative genera are shown, together with E values, indicating the numerical expectation that the alignment with the reference sequence has arisen by chance. Support values (in red) were determined by bootstrap analysis (Additional file 1o). Branch lengths are proportional to number of amino acid substitutions per position. Additional file 2o gives branch length values and confidence limits from the PROML outfile.

As well as representing a destination for pigments and their catabolites, the vacuole has complex functions in intracellular turnover, lytic processes and defence against biotic invasion. See2 (UniProt CAB64545) is the product of a *Zea *gene originally identified amongst those upregulated during flag leaf senescence [82]. It is a representative of the VPE group of protein-processing legumains and may function as part of a proteolytic cascade, rather in the manner of the caspases involved in apoptosis [83]. The *Arabidopsis *homologue (At4g32940) is most strongly expressed in senescent and cauline leaf, with some significant signal in petal, sepal, xylem and cork (Figure 2). BLAST identified similar proteins in angiosperms, conifers, mosses and green algae (Figure 19). A distantly diverged group comprises marine myxobacteria, diverse vertebrate and invertebrate taxa.

### Cluster analysis of senescence-related genes

**Figure 20:**
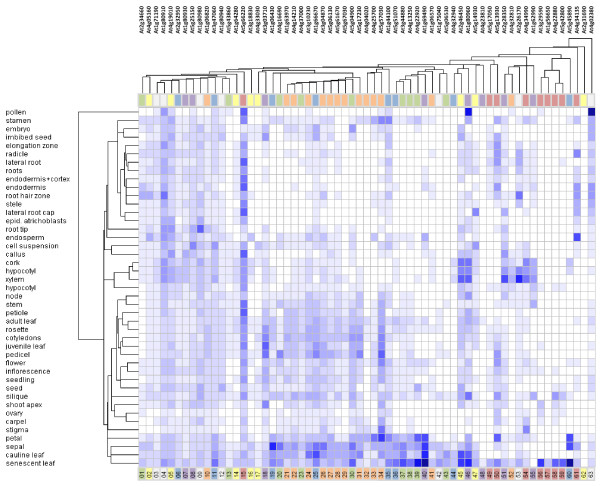
**Genevestigator heat map of *Arabidopsis *genes with functions and transcription patterns related to senescence, clustered by tissue and expression profile according to transcript abundance**. Heat maps represent expression level in Affimetrix^® ^arrays as shading intensity computed in Genevestigator according to the MAS5 normalization algorithm [192]. Genes are colour-coded by broad functional categories and listed in order as shown in Figure 1.

In the evolution of the senescence program, genes would be expected to show a degree of coordinate expression in groupings that reflect phylogenetic trends. Similarly, a consequence of the differentiation of plant tissues and organs from ancestral structures is the sharing of common senescence programs. We used the Genevestigator Clustering Analysis tool to look for relationships between a collection of *Arabidopsis *genes comprising those listed in Table 1 and a further group that includes the results of a search of the TAIR database for senescence-related genes. Figure 1 lists a total of 63 genes, each of which is assigned to a colour-coded broad functional grouping (no colour indicates an unknown, unclassifiable or hypothetical protein). The Genevestigator heat map shown in Figure 20 clusters these genes according to profile and tissue, in the order left to right in which they are listed in Figure 1. The distribution of colour codes indicates blocks of genes with common functions and coordinated expression patterns. Tissues with similar complements of expressed genes are hierarchically arranged close to each other. The gene and tissue associations presented in Figure 20 are suggestive of developmental and evolutionary relationships that are considered further in the Discussion section below.

## Discussion

**Figure 21:**
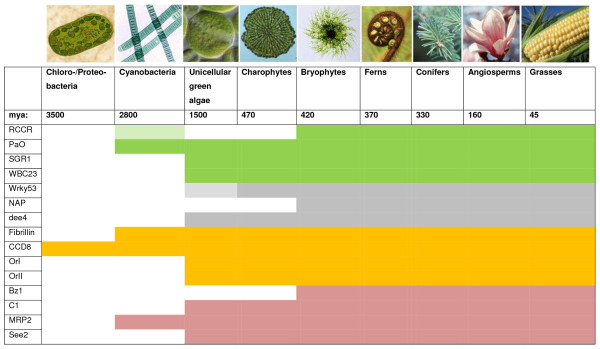
**Proposed evolutionary timeline for senescence-related genes discussed in the present study**.

The present account of the evolutionary origins of plant senescence seeks to follow the major features of the syndrome in angiosperms through the lineage of organisms leading from the earliest phototrophs, as revealed by analysing sequence relationships among key proteins. Additional File 3 summarises innovations in the evolutionary time-line that are relevant to the construction of a senescence program integrated into plant development and adaptation, and represents a framework for the discussion that follows. Figure 21 relates this time-line to the phylogenetic origins of the senescence-related genes analysed in the present paper. The foundation of the argument developed here is that a core of primary metabolic processes concerned with building and maintaining the photoreceptors of the earliest prokaryotes has been invested with successive layers of adaptive metabolic and regulatory circuitry as factors in the evolution of complexity in trophic status, life-cycle, anatomy, morphology, adaptation to new environments and biotic and abiotic challenges.

### Pre-endosymbiotic phase

The Archaeozoic and Mesoproterozoic eons lasted from 3800-1000 mya. Although the detailed phylogeny of this period is difficult to infer from molecular analysis of contemporary prokaryotes [84], it is probable that the fundamental design of the photosynthetic apparatus was established early, in the form of pigment-protein photoreceptors driving an ATP-generating proton pump [13]. The ubiquity of genes for isoprenoid synthesis [49] is evidence of their ancient and fundamental role in light interception and photochemical quenching [45,85,86]. Genes for (bacterio)chlorophyll biosynthesis have been used to create well-rooted trees leading back to the putative earliest photosynthetic organisms [15]. Assembling complexes requires translational regulation [87] and efficient facilitated shape-matching [88], to coordinate the supply of structural elements. In modern autotrophs a major factor in achieving stoichiometry is post-transcriptional culling of excess unmatched components [89-91]. Thus in mutants deficient in pigment or protein components of photosystems, most of the genes responsible for building the complexes are transcribed and translated normally, but because they cannot make stable assemblages the proteins are degraded by fidelity-testing peptide hydrolases. For example, Fu et al. [92] showed that the cyclophilin AtCYP38 is required to stabilise thylakoid complexes in *Arabidopsis*: mutants deficient in CYP38 make normal amounts of photosystem structural components but these fail to assemble correctly and are turned over. Significantly, the CYP38 gene (At3g01480) is strongly down-regulated in senescing compared with mature and juvenile *Arabidopsis *leaves (data not shown). Xiong [15] described how a functional photosynthetic apparatus could have evolved in the earliest prokaryotes by multi-staged recruitment of reaction centre and antenna proteins with previously diverse evolutionary histories and functions. We consider that disassembly of complexes is correspondingly hierarchical in origin. Phylogenetic analyses of plant proteases [93] and of proteasome components and organisation [94] shows convincingly that the catabolic machinery for protein turnover is as ancient as the Chlorobacteria and Proteobacteria at the base of the evolutionary tree.

Charge-separation is hazardous for pigment-protein reaction centres and here again there will be a requirement for turnover systems to maintain functioning structures [95]. Genes specifying cellular systems for turning over photoreceptor complexes may therefore be expected to be ancient and we hypothesise that the distribution of protein sequences implicated in some features of angiosperm senescence represents evidence for continuity back to the pre-endosymbiotic origins of green plants. Studies of their function in chloroplast senescence suggest that Sgr and PaO are components of a machine that destabilises thylakoid pigment-proteolipid complexes by removing chlorophyll and exposing the associated proteins to attack by peptide hydrolases [19,65,96]. Plastoglobules play a part in this process, receiving hydrophobic products released from dismantled membranes, including carotenoids, phytol from chlorophyll and products of lipolysis [55,97]. The structural similarity between the PaO- and Fibrillin-like proteins of prokaryotic phototrophs and the corresponding proteins of angiosperms (Figures 4, 13) supports the proposition that these components of the machinery for unpacking pigment-protein complexes are ancient.

### Endosymbiotic associations in the origin of eukaryotic plants

The origin of the chloroplast as a cyanobacterium-protozoan symbiosis occurred, according to most estimates, around 1500 mya or earlier [98], though there is an alternative view placing it as late as 600 mya [23]. The primary event soon gave rise to two lineages, the glaucophytes and the green plant/red algal group. Mixotrophy and opportunist organotrophy were probably well established in the earliest phototrophic prokaryotes and, in response to increasing competition for nutrients, vestiges of facultative trophic behaviour have persisted throughout the period from the enslavement of the endosymbiotic ancestors of eukaryote organelles to the plastid differentiation network of modern angiosperms (Figure 12; [24]). The metabolism of green cells of angiosperms changes from auto- to hetero-trophic as the photosynthetic apparatus is remodelled during the transdifferentiation of chloroplasts into chromoplasts or gerontoplasts. Light is a declining source of energy in these cells and respiratory processes become increasingly significant [99]. Fruit ripening in many species, and leaf senescence in a few, is accompanied by a respiratory burst (climacteric; [100]). The amino acid products of protein recycling are subjected to transamination, deamination and amide synthesis to provide carbon skeletons for respiration while salvaging nitrogen in a transportable form [101]. A gene encoding an amino acid permease is upregulated when *Auxenochlorella protothecoides *is transferred from auto- to hetero-trophic culture conditions; the *Arabidopsis *homologue is also upregulated in senescing leaves (Figure 2). The exact identity of the protease(s) responsible for releasing amino acids from photosynthetic proteins is not agreed [102]. A number of plastid-located proteases are known [90], amongst which at least four (ClpP3, ClpD, FtsH7 and FtsH8) are upregulated in senescence (Figure 20). BLAST searches show that highly homologous sequences to all four proteins are widely distributed across taxa including phototrophic and non-phototrophic bacteria. There is evidence that Sgr is essential for making chlorophyll-binding proteins available to proteases [9,69,89]. Sgr is not detectable in photosynthetic prokaryotes, although Sgr-like sequences are present in some firmicutes, possibly as the result of horizontal transfer (Figures 6, 7). We suggest that Sgr was recruited to the system for dismantling pigment-proteolipid complexes when subcellular topology became more complex with the appearance of the eukaryotic condition. This in turn suggests that protein mobilisation and colour change in tracheophyte senescence have their origins in the intraplastidic turnover of pigment-proteins related to regulated assembly of macromolecular complexes and trophic flexibility.

An alternative, or additional, mechanism for proteolysis in senescence involves exchange of protein substrates and/or proteases between plastids and vacuoles in as-yet uncharacterised vesicles [103-105]. This would assign a role in senescence to vacuolar proteases such as SAG12 ([106]; Figure 20) and to components such as See2 (Figures 2, 19) that may trigger proteolytic cascades [83,107]. Martinez and Diaz [93] have studied the evolution in plants of C1A papain-like cysteine endopeptidases (SAG12 is one of these) and the C13 VPE-like legumains (includes See2) that may activate them. C1A proteases are present in angiosperms, mosses, *Volvox*, *Chlamydomonas *and *Ostreococcus*. Martinez and Diaz [93] report that *Ostreococcus *has no C13 VPE sequence, but the NCBI BLAST search reported here has produced two hits: one (UniProt Q00VF5) from the published study of *O tauri *by Derelle et al. [108], the other from the US DOE Joint Genome Institute *O lucimarinus *database (B Palenik et al. unpublished submission 2007). In both cases the E value for alignment with See2 is better than 1e^-13 ^and for the C13 peptidase superfamily conserved domain E scores were of the order of 1e^-80^. We conclude that VPE-like proteases are present in *Ostreococcus *(Figure 19).

An ancient function of the cell vacuole is to act as an autolytic body in cell death. 'Metacaspases' (a name for vacuolar proteases that may function like the caspases of apoptotic animal cells but which have little or no homology to them) have been implicated in a cell death program in diatoms [109]. It is doubtful that autolysis plays a part in the initiation and execution of the senescence program in angiosperms [110] but it may be critical in the terminal phase of tissue development when senescence is ending – for example in vascular tissue where the cells are dead at maturity [111,112] or as a cauterisation measure in senescent mesophyll [106,110]. We speculate that the progression from *Ostreococcus *to *Volvox *to *Chlamydomonas *shown in Figure 19 may signify the appearance and development of a capacity for proteolytic processing by See2-like VPEs during the period of green alga evolution associated with a new role for the vacuole in organelle protein turnover.

The auto- to hetero-trophic switch induced in *Auxenochlorella protothecoides *by light and nitrogen limitation is associated with excretion of bilin products of chlorophyll catabolism [28]. It is not known whether such catabolites have antibiotic or allelopathic properties, though they are certainly highly photodynamic. Also unknown is the transporter that moves bilins across the cell membrane. ATP-dependent transporters that export chlorophyll catabolites from plastids to cytosol and from cytosol to vacuole have been identified in *Arabidopsis *and the corresponding genes (At5g06530 encoding the plastid envelope transporter, At2g34660 encoding one of the tonoplast MRP2 transporters) are upregulated during senescence (Figure 2). BLAST identifies algal sequences with similarity to AtMRP2, and comparisons of domain structure suggest common functions (Figure 18). However the sequence of the AtMRP2 protein has higher similarity to that of AtMRP4 than to its *Ostreococcus *homologue. Both AtMRP2 and AtMRP4 proteins have the conserved domain structure of full-molecule ABC transporters, but the former is located in the tonoplast whereas the latter is a plasmalemma component [73]. Such an MRP in algae, with similarities to both MRP2 and MRP4, would have the expected properties of the transporter responsible for ejecting chlorophyll catabolites from the cell. Subsequently, it is conceivable that tonoplast and plasmalemma transporter localisations and functions diverged as the participation of the vacuole in chlorophyll catabolism became established as part of a novel physiological module added downstream of bilin formation when multicellular plants emerged onto the land [113].

### Development of multicellularity

The Ordovician-Silurian period (450-410 mya) saw the rise of the charophytes, which are considered to link the unicellular chlorophytes with the first land-plants. The lack of sequence data for members of the Charophyta is a major gap in the phylogenetic record, but it is possible to identify features within charophyte algae likely to be relevant to senescence evolution, notably the elaboration of multicellular morphology, branching, 3-D cell division and interconnection of cells by plasmodesmata [43]. A key genus is *Coleochaete*, which includes branching filaments and thallus-like parenchymatous forms. Molecular phylogenies based on chloroplast and ribosomal sequences put this group close to the evolutionary line between unicellular chlorophyceans and bryophytes [114].

The presence of lignin-like compounds in *Coleochaete *[115] is evidence of a capacity for phenylpropanoid metabolism which would have been the ancestral basis for the elaboration of lignified vascular and mechanical issues, and later the rise of flavonoid pigmentation, in the land flora. Long-distance symplastic transport was already well established in green algae, including charophytes, and is observed in bryophytes alongside the beginnings of vascular translocation structures [40]. Long-range nutrient redistribution, from green cells in which the balance of turnover favours net dismantling of macromolecules and cell structures, is thus anatomically feasible in the charophytes.

The development of multicellular two- and three-dimensional forms changes the relationship between the plant body and the external medium. Direct exchange of materials across the plasmalemma has to be subordinated to the structural and physiological requirements of the tissue. It seems likely that this was the impetus for the evolution of more complex roles for the cell vacuole, which took on the status of 'inner space', in a sense adopting the role of a remnant of the ancestral aquatic milieu inside each vegetative cell. Extensive studies of the vacuoles of *Chara *and *Nitella *show them to perform sophisticated functions in ionic regulation, hydraulic maintenance of tissue morphology and turgor-driven cell wall growth [116-118]. They are also highly lytic, sequestering a range of proteases and other acid hydrolases [119-121]. The vacuole of charophytes thus possesses, or is en route to developing, most of the characteristics of the subcellular compartment that functions in angiosperm growth and senescence [122].

### Plants invade the land

Green plants made landfall in the Silurian-Jurassic period, more than 410 mya. In this new and hostile environment, desiccation was avoided and ionic homeostasis maintained by the early development of an all-enveloping epidermal and cuticular 'space suit' [123] and a water-transport system [40,124]. The capacity for lysigeny and schizogeny was established in the first land plants, in connection with both the differentiation of vascular systems and the shedding of parts [124,125]. Along with mechanisms for smoothing out extreme variations in water-supply, it was necessary to establish physiological and developmental adaptations to deal with wide fluctuations in the inputs to photosynthesis. Many of the adjustments to the primary events of light capture and CO_2 _assimilation within chloroplasts are regulated by systems present in *Chlamydomonas *and other unicellular chlorophyceans [45]. Atmospheric CO_2 _concentration 410 mya is believed to have been around 3000 to 4000 ppmv [126]. This, combined with exposure to light fluxes no longer attenuated by the water-column, has led to the suggestion that the move to the terrestrial environment stimulated carbon fixation and utilisation to the point of incontinence – in the words of Harper [127] 'the green plant may indeed be a pathological overproducer of carbohydrates'. It allowed plants to develop profligate, throw-away lifestyles based on lysigeny and schizogeny on a vast scale. For example, primitive trees such as *Archaeopteris *(359-349 mya) probably controlled canopy morphology through abscission of lower megaphylls [128]. Abscission may have also been important in dehiscence of sporangia even before it became significant in vegetative shaping of plants [128], and a deciduous *Glossopteris *flora was already well established in Gondwanaland during the Carboniferous era [129]. Preserved senescent plastids have distinctive features that enable them to be identified in fossilised material [130]. Thomas and Sadras [131] discussed senescence as a strategy evolved by early land plants to deal with the promiscuous productivity of a photosynthetic machinery optimised for the relatively stable ancestral aquatic environment.

By leaving the water, plants lost the option of expelling unwanted or aggressive metabolites directly from individual cells into the outside environment. To a great degree the cell vacuole has taken over as the destination for such compounds. Chlorophyll degradation during the transdifferentiation of chloroplasts to gerontoplasts (Figure 12) illustrates this change in the relationship between cells and their environment. The catabolic pathway of chlorophyll in land plants is organised so as to limit photodynamic damage by free pigments. Chlorophylls and their coloured derivatives must be moved around and interconverted within strongly quenching microenvironments inside the plastid until the risk of photodamage is finally removed by opening the tetrapyrrole ring [9]. Sgr, PaO and Fibrillin are important participants in this phase of the process. Algae like *Auxenochlorella *expel RCC, the product of the PaO reaction, directly into the medium, possibly via an MRP2/4-like transporter as discussed above. Land plants, on the other hand, have requisitioned a detoxification pathway to redirect the products of chlorophyll catabolism to the cell vacuole. The two activities that make the link between the evolutionarily ancient first section of the catabolic pathway and the cytosolic-vacuolar steps are RCCR and the envelope transporter WBC23. The presence of the latter in *Chlamydomonas *and *Ostreococcus *is identified by BLAST; BLAST analysis also records hits on similar choanoflagellate and cnidarian sequences (Figure 8), suggesting that an ABC transporter similar to that which moves straight-chain bilins across the plastid envelope was present at or before the evolution of the chloroplastic endosymbiont.

Using site-specific mutagenesis methods, Pružinská et al. [132] have shown unequivocally the in vivo role of RCCR in chlorophyll catabolism; but the enzyme appears to have some other function too, concerned with mitochondrial participation in programmed cell death [133]. The occurrence of RCCR-like sequences is consistent with the appearance of this enzyme at the point of land plant evolution [17], though there are BLAST hits in the cyanobacteria (Figure 3). Why is there a discontinuity in the distribution of RCCR? Although there is a considerable step up in E value between the land plants and cyanobacteria, the *Nostoc *and *Anabaena *proteins have all the structural features of RCCR when examined by the conserved domain tools of NCBI BLAST and Pfam. BLASTing against CYANOBASE (the Cyanobacteria genome database) revealed weak similarity with phycoerythrobilin ferredoxin oxidoreductases, which may conceivably have been the distant ancestral sources of plant RCCRs. BLASTing the *Nostoc punctiforme *genome database reveals two genes located side-by-side (Npun_R3279 and Npun_R3277). They are highly similar and may represent a duplication. Pfam recognises both proteins as possessing the conserved domains of RCCR. Global transcription data are available for *Nostoc *and show that 3277 is strongly expressed during hormogonia differentiation but not in other developmental stages. By contrast 3279 was down-regulated during hormogonia differentiation [134]. *Nostoc *is an extracellular symbiont or endophyte in the hornwort *Anthoceros *[135], but an intracellular symbiont in the angiosperm *Gunnera *[136]. The hormogonium phase is the one that colonises the host. The available *Anabaena *genome (*A variabilis*) has only one RCCR-homologous sequence. *Anabaena *is symbiotic with the water fern *Azolla *[137].

It is significant that the only cyanobacteria known to have RCCR homologues (functional at the transcript level in the case of *Nostoc *at least) are those that form symbioses with green plants in tissues which are exposed to light. In these associations (especially the intracellular symbiosis), the normal option of excreting RCC into an aqueous medium would not be available. We speculate that this is the reason for the anomalous occurrence of RCCR genes in symbiotic *Nostoc *and *Anabaena*. The source of these genes remains problematical. Did land plants acquire RCCR by gene transfer between a cyanobacterium and a hornwort-like early colonizer host; or (more likely, since there is no obvious trace of the gene in other cyanobacteria) did a cyanobacterial symbiont pick up the gene from its host? The possibility of gene transfer between hosts and symbionts as a factor in the adaptation of early land plants to the new environment merits further study.

RCCR converts the phototoxic chlorophyll catabolite RCC into pFCC, which the envelope transporter WBC23 exports to the cytosol. The subsequent reaction sequence – conjugation followed by sequestration in the vacuole – is the usual means by which intracellular waste products or xenobiotics are detoxified [138,139]. The tonoplast transporter MRP2 was discussed above.

Beerling [140] has proposed that the high atmospheric CO_2 _concentrations at the time when plants invaded the land persisted for 40–50 my, until the late Devonian, when a steep fall in CO_2 _drove the evolutionary transition from microphyllous plants (psilopsids, lycopsids, bryophytes) to the macrophyllous and euphyllous ferns, conifers and angiosperms. Beerling points out that the genes for leaf morphogenesis were to a large extent already present in clubmosses, spikemosses and quillworts and proposes that high CO_2 _constrained the expression of this morphogenetic potential. The elaboration of megaphyllous laterals from the Carboniferous onward led directly to the evolution of the specialised reproductive and propagative structures of the angiosperms according to long-established ontogenetic principles [39,141]. Although light, carbon and (with qualifications) water may have been superabundant, mineral nutrients are likely to have been a limiting factor in early terrestrial ecosystems. Nitrogen in particular is released in only very small amounts from the weathering of rocks, though it is possible that biological N_2 _fixation by (cyano)bacteria and archea and nitrogen oxide fertilization by electrical storms could have contributed to the formation of skeletal oligotrophic soils as far back as 1200 mya [142]. The requirement for parsimony in the internal nitrogen economy of early land plants would have favoured the development of a number of structural, developmental and physiological traits contributing to the adoption of an integrated senescence program [38,142].

Efficient internal nitrogen recycling requires cell integrity and metabolic regulation in the senescing source tissue, a directional translocation system connecting the source to the developing sink, means of signalling supply and demand status between the different regions of the plant body and a morphogenetic context that maintains structural and functional fitness [40,143,144]. Development of the thalli of liverworts clearly includes the capacity for senescence-like terminal changes (see, for example, Koeberl and Maravolo; [145]). Eckstein and Karlsson [146] showed the recycling of nitrogen from senescing to young ramets in mosses lacking a specialised vascular system. This suggests that source-sink integration of senescence into development was well established before the evolution of tracheophytes [40]. A search of the JGI *Physcomitrella patens *online database at the time of writing yields more than 330 hits with high sequence similarity to senescence-related genes in *Arabidopsis*, rice and *Medicago*, and proteomics studies, such as the analysis of responses of the *P patens *phosphoproteome to cytokinin treatment described by Heintz et al. [147], also identify gene products that may have a function in the developmental integration of senescence in this species. Highly similar homologues of all the representative senescence-related proteins analysed in the present study have been identified within the bryophytes by sequence-based analysis (Figure 21). We conclude that the genetic potential for expressing and regulating the senescence syndrome and deploying it as an adaptation to oligotrophic conditions was well established in the earliest land plants.

The senescence program is integral to strategies for dealing with the biotic and abiotic stresses of the terrestrial environment. Wrky53, member of a family of regulatory genes that diversified greatly with the evolution of the tracheophytes ([74]; Figure 9), encodes a transcription factor that functions both in senescence pathways and in a network of stress responses [148]. Wrky53 interacts with more than 60 genes of various kinds, including those for other transcription factors and for the senescence-related cysteine endopeptidase SAG12 [149]. Wrky53 is induced by H_2_O_2_, and autoregulates its own synthesis by feedback inhibition. Salicylic acid and jasmonic acid, via the regulatory protein ESR, control Wrky53 functions in senescence and resistance responses to pathogens [150]. Acclimation and adaptation to environmental stresses is often mediated by redox sensing. The stress-activated transcriptional cofactor NPR1 exists in the cytoplasm in the form of disulfide-bonded oligomers. A change in redox conditions results in reduction of the intermolecular disulfide bonds and relocation of monomeric NPR1 to the nucleus where it activates genes [151]. Miao et al. [149] reported that At4g26120, a gene encoding an NPR1-like protein, is a target for Wrky53 binding. The corresponding protein sequence shows better than 99% identity with that encoded by At1g64280, transcription of which is enhanced in senescence (Figure 20). Other genes listed in Figure 1 and profiled in Figure 20 with which Wrky53 interacts include At4g33030 (sulpholipid synthase), At5g45890 (SAG12 protease) and At5g14930 (SAG 101 lipase). It may be significant that the latter gene clusters immediately adjacent to Wrky53 in the Genevestigator diagram (Figure 20).

### Tracheophytes, angiosperms and co-evolution

Angiosperms appeared in the Jurassic period, 150 mya. Carpels, flowers and tectate pollen occur in the fossil record at around this time and within 30 my angiosperms had become the dominant component of global floras. Before evolution of the angiosperms the colour world of vegetation would have consisted largely of greens, yellows and browns, much as it does in modern conifer-dominated forest ecosystems. A rapid increase in the profusion of natural pigmentation accompanied the Cretaceous explosion in angiosperm evolution and was likely to have been an adaptive response to a combination of abiotic and biotic factors [152,153]. Radiation of the first angiosperms into a wide range of new and often hostile environments was made possible by physiological and morphogenetic innovations or consolidations including, for example, the development of flexible photosynthetic mechanisms and the proliferation of architectures and life-forms [152,154]. Climate change may also have been a driving force at the onset of angiosperm diversification, since a transition between icehouse and greenhouse conditions occurred at this time [155].

Photoinhibition and the damaging effect of excess light energy within assimilatory tissues is a common factor in the experience of abiotic stresses [95,156]. The ancient role of carotenoids in the dissipation of excess light energy within the photosynthetic apparatus [45] assumed new significance in land plants as they moved out into ecological niches in which the abiotic environment was increasingly subject to wide fluctuations that differentially affected the capture and utilisation of light energy [157,158]. Amongst the carotenoid-related stress responses shared with senescence is the photoprotection role of Fibrillin [159]. Upregulation of Fibrillin expression in senescence (Figure 2) is consistent with the requirement for the maintenance of photoprotection during chlorophyll catabolism and protein mobilisation within the transdifferentiating plastid [9].

Coevolution with pollinators and dispersers is an important factor in establishing carotenoid- and phenylpropanoid-based pigmentation of floral organs and propagating structures such as seeds and fruits [153,160-163]. The interaction between the colours of plant parts and the animal visual system is an example of extreme convergence in evolution, in the sense that the isoprenoid-protein light receptor structures and metabolic systems in the earliest photoautotrophs (evolved to exploit the main central part of the visible spectrum) show remarkable gene and protein sequence conservation through to both the carotenoid biochemistry of angiosperm organs and the eyes of the animals that interact with them. For example the highly conserved carotenoid-metabolising enzyme CCD8 (Figure 14) is a member of the same superfamily (RPE65) as the membrane proteins that bind retinal pigments in the eye. In contrast with the extremely ancient biochemistry and functions of isoprenoids, molecular phylogeny identifies flavonoids, including the hydrophilic vacuolar pigments of leaves, flowers and fruits, as innovations of the land plants (Figure 16, Figure 21; [164]), and their diversification as a factor in angiosperm radiation [153,162,165]. The evolutionary origin and significance of red anthocyanin coloration in senescing leaves are subjects of much current debate [8,59,166]. Modelling, and some experimental evidence, supports the idea of coevolution with herbivores, in which foliar anthocyanins act as signals to insects and other predators [167]. Alternatively, or additionally, anthocyanin accumulation may be a plant response to, or insurance against the effects of, abiotic stresses such as extremes of, or imbalances in, light, temperature, water availability or nutrient status [168]. We shall return to the question of the evolutionary relationship between the pigments of senescing foliage and the colours of flowers and fruits.

According to Ruddiman [169] the present era is the Anthropocene (a term coined by Crutzen and Stoermer; [170]) and dates from the origins of agriculture in human evolution, more than 8000 years ago. Human activity has intruded on plant evolution during the Anthropocene not only by subjecting it to environmental and climatic perturbations but also, and more directly, through selection, crop improvement and biotechnology. The domestication syndrome comprises, amongst a range of traits, annual growth habit, hypertrophied grains and foliage, rapid seedling establishment and high harvest-index [171]: all of which are influenced by, and influence, the timing and execution of senescence programs [172]. The senescence-associated NAC transcription factors ([41]; Figure 10) illustrate the point. Ooka et al. [173] analysed the NAC family of *Arabidopsis *and rice and placed At1g69490 and three other closely related sequences (At1g61110, At3g15510, At1g52880) in the NAP subgroup of Group I. Genevestigator expression profiling shows that each of the four AtNAPs in this family has a distinctly different tissue specificity: highest abundance of At1g69490 transcripts is in senescing leaves, with petals and sepals also giving a strong signal (Figure 2). At1g52880 expression is most intense in seed and fruit tissues, with some signal in senescing leaves; At3g15510 transcripts are most abundant in cork and xylem; whereas At1g61110 expression is confined to flowers and stamens (data not shown). At1g69490 is the top hit when the *Hordeum *senescence-associated NAM protein sequence is BLASTed against the TAIR8 protein dataset. Sequence alignment also places rice ONAC010 in the NAP subgroup, and BLASTing the rice sequence hits both the *Hordeum *and *Triticum *senescence-related NAM proteins with E values of better than 3e^-100^. A transcriptomic study by Hirose et al. [174] showed that ONAC010 expression in rice leaves is suppressed by treatment with the anti-senescence hormone zeatin. Divergence of function and structure is evidence of the plasticity of genes of the NAP/NAM family under natural and, potentially, human-mediated selection. Functional alleles of the NAM-B1 gene, encoding a transcription factor that accelerates senescence and increases nutrient remobilization, occur in ancestral wild wheat. Modern wheat varieties have been selected for delayed senescence, a trait correlated with enhanced grain yield [172]. They carry a non-functional allele of NAM-B1 [41]. Many further examples of human intervention to alter the senescence and ripening patterns of domesticated plants (with consequences for pigmentation or productivity or both) are discussed in [175-177].

### Relationship of ontogeny and phylogeny in the evolution of senescence

**Figure 22:**
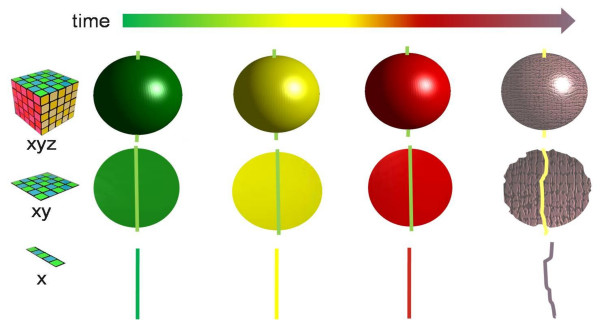
**Ontogenetic and phylogenetic interrelationships of plant organ senescence**. The diagram represents morphogenetic relationships between one-, two- and three-dimensional organ structures (for example, linear axes, laminae and fleshy fruits respectively) and the time-related progression through senescence/ripening to death signified by characteristic changes in pigmentation.

During the present discussion we have moved freely across the full evolutionary range of taxonomic groupings and between cells, tissues and organs and their genes, regulators, enzymes, transporters, pigments and metabolites. To justify treating processes and structures, as well as genes and proteins, as homologues, we propose here a simple unifying developmental scheme. Following the principles of morphological and allometric transformation first described in 1917 by d'Arcy Thompson (revised edition 1992; [178]), a linear branch- or microphyll-like structure may evolve by 'rubber sheet' distortion into a two-dimensional lamina (a leaf or floral part for example), which in turn can be expanded in a third dimension to produce a fleshy spheroidal structure – a fruit, say (Figure 22). By adding a developmental time dimension to this figure, the progress of senescence may be represented by a sequence of pigmentation changes, green through yellow to red and finally the post-senescence transition to cell death accompanied by non-physiological darkening or bleaching. The result (Figure 22) is a spatial-temporal grid across which the aerial structures of multicellular green plants may travel freely during development or evolution. The grid itself is plastic and deformable so that particular structural or temporal stages are curtailed, extended or even subject to reversion, thereby giving rise to the profusion of forms and senescence behaviours seen in the land flora.

This schematic description of the homologous interrelationships of organ senescence has important implications when considered together with the molecular relationships described in the present study. It is clear that the competence to transdifferentiate chloroplasts into gerontoplasts was already well established in the bryophytes and ferns, whereas chromoplasts appeared later when coevolution with the visual systems of interacting animals began to drive diversification of the colours and morphologies of reproductive structures (Additional File 3, Figure 22). In this regard it is significant that genes for Fibrillin, OrI and OrII, which originated phylogenetically long before chromoplasts had evolved (Figures 13, 15, are close to each other when clustered by expression profile (Figure 20, tracks 32, 27, 28). We argue, therefore, that there is a direct relationship between the phylogenetic precedence of gerontoplasts over chromoplasts on the one hand and their corresponding ontogenies on the other [113]. This implies that leaves which lose their chlorophyll and reveal and/or accumulate non-green pigments (carotenoids in all groups, anthocyanins arising with the cryptogams and angiosperms – Figure 21) are the evolutionary progenitors of the highly coloured flowers, fruits and seeds of dicots and monocots. It means that, phylogenetically and ontogenetically, the pigmented sepals, petals and dispersal structures of angiosperms are essentially heterochronic senescing leaves [110].

Moreover, if the developmental sequence of the two-dimensional structure in Figure 22 represents the ontogeny of a leaf and a homologous perianth structure such as a petal, then it is *formation and pigmentation *of the floral organ rather than the subsequent fading and death of the structure that expresses the modified senescence program. This view is not widely accepted (see [3,179]), but receives some support from the literature (for example [180-182]) as well as from genomics. When senescence-related transcription profiles are used to cluster tissues and organs according to similarity of expressed gene complement (Figure 20), senescent leaf, petal, sepal and other floral parts form a clear grouping. On the other hand xylem and cork, tissues in which developmental cell death is an essential part of the differentiation program, are relatively distant from the leaf and floral clades (Figure 20), which is consistent with the argument that foliar senescence and homologous processes such as perianth development should be classed as transdifferentiation phenomena distinct from terminal cell death events [110].

The clustering patterns of Figure 20 provide further insights into the possible nature and origins of plant senescence and its regulation. It is striking that 11 of the 14 genes for isoprenoid metabolism analysed form a group between tracks 21 and 34. A coordinated unit of genes determining carotenoid functions in transdifferentiating plastids is suggested. Similarly, of 9 phenylpropanoid genes, 8 cluster between columns 49 and 61, indicating an integrated regulatory grouping. PaO, chlorophyll b reductase and Sgr are immediately adjacent to each other (tracks 37–39) and near to WBC23 (track 43). The remoteness of chlorophyllase from this cluster (column 13) may reflect growing opinion that the enzyme product of the corresponding gene (At5g43860) may not function in chlorophyll degradation in vivo [183]. The status of phaeophorbidase (column 20) in physiological chlorophyll catabolism is also uncertain. Like RCCR, which is nearby (track 23), no algal homologue has been detected [33], suggesting it too may be a relatively recent recruit to the pathway. The isolation of MRP2 (track 1) from other components of the chlorophyll breakdown pathway may reflect its variety of functions and ancient evolutionary origins. The associations revealed by cluster analysis (Figure 20) and the phylogenetic diversity of senescence-related genes (Figure 21) are consistent with the proposition that senescence is more a timetabled network than a single executable program [5]. Senescence has its evolutionary origin in a core of metabolic processes concerned with building and rebuilding photosynthetic complexes. Other biochemical, cellular, integrative and adaptive systems became accreted on this armature as the evolving plant encountered new environmental and developmental contexts. Current systems biology approaches [184] are leading towards the idea of senescence as a complex of reticulated gene interactions with nodes and hubs and away from the older conception of a master switch from which subsequent events propagated [5]. The evolutionary perspective is entirely consistent with such a system network model of plant senescence and its control.

## Conclusion

In recent times the quantity and quality of DNA sequence information from plants across the taxonomic and evolutionary range has increased greatly. At the same time our understanding of the molecular and cell biology of plant senescence has developed to the point where we can assign functions to some enzymes and regulatory proteins. In the present paper we have taken the first steps towards a synthesis that allows us to frame, and begin to test, hypotheses about the evolutionary origins of angiosperm senescence, its control and integration into plant development and adaptation. Because major gaps remain in the taxonomic spread of sequenced species, and the assignment of functions to senescence-associated genes, the arguments presented here include a number of conjectural interpolations and extrapolations. For example, it is something of an article of faith for molecular evolution studies to consider a modern member of an ancient phylum (the lycopod *Selaginella*, for example) as representative of the genomic status of its newly-evolved ancestral form. Many more sequences from many more species, particularly covering the span between the cyanobacteria and the angiosperms, are needed before this belief can be held with full confidence. For the specific case of plant senescence, the scarcity of sequences from ferns and charophytes is a particular limitation. Even so, the present study has allowed a feasible scheme to be proposed that accounts for much of the available information on senescence-related genes, their functions and interactions.

## Methods

### Databases accessed

The following database sources were used in this study.

TAIR – the *Arabidopsis *Information Resource



TIGR *Arabidopsis *database



SwissProt and other protein databases accessed through NCBI Entrez

Genbank and other nucleotide databases accessed through NCBI Entrez

Conserved domains accessed through NCBI Entrez



UniProtKB protein knowledgebase



JGI Integrated Microbial Genomes (IMG) system



JGI *Chlamydomonas reinhardtii *v 3.0 BLAST



CYANOBASE



JGI *Physcomitrella patens *online database



Gramene data resource for comparative genome analysis in the grasses



Tree of Life web project



### Sequence alignment and structural comparison tools used

BLAST, including protein BLAST (blastp), nucleotide BLAST (blastn, megablast), blastx, tblastn, tree viewer and BLINK precomputed BLAST



EBI FASTA fast protein comparison



EBI ClustalW2 sequence alignment tool



GENEDOC sequence alignment editor



EMBL SMART domain architecture tool



Pfam protein domain database



### Creating and displaying protein sequence trees

All protein trees reported here were generated using, as appropriate, BLASTP and FASTA for local alignments, ClustalW2 for global alignment, and TREE-PUZZLE, PUZZLEBOOT and the PHYLIP Phylogeny Inference Package [185,186] for phylogeny and bootstrap analysis.

Protein sequence trees were constructed from amino acid sequence data using PROML (Protein Maximum Likelihood Program) in PHYLIP 3.62 for inferring evolutionary trees. PHYLIP (current version 3.68, released in August 2008) is distributed by the author, J Felsenstein, Department of Genome Sciences, University of Washington, Seattle. . Input files for PROML, consisting of multiple sequence alignments, were created using ClustalW2. The Jones-Taylor-Thornton model was used to compute probabilities of changes between amino acid sequences.

For all amino acid sequence data sets, PROML was run with three different settings for the R (rate variation among sites) parameter: Gamma distribution [187], Hidden Markov Model (HMM [188]) and constant rate of change. The Log Likelihood (Ln) values of these three approaches for each set of protein sequences studied are summarised in Additional File 4. Although there were some minor variations in Ln values using the different approaches for estimating the among-site rate heterogeneity, the final tree conformations were the same regardless of which approach was used. Thus the among-site rate variation did not have significant influence on the topology of trees produced in this study.

Bootstrapping analyses were carried out on all datasets to measure how consistently the data support given taxon bipartitions. The bootstrapping majority-rule consensus tree and support values were generated through the use of SEQBOOT, NEIGHBOR and CONSENSE programs in PHYLIP, and PUZZLEBOOT . High bootstrapping values mean uniform support; a support value of less than 50 is treated as giving no support. Support values generated in this study are presented in Additional File 1 and indicated in each tree figure. Tree branch lengths, generated using PROML with Gamma distribution, are summarised in Additional File 2. Bayesian analysis using the TOPALI  package was also performed on all datasets and the output of support values from this analysis is consistent with the support values presented in this study.

To estimate the degree of rate heterogeneity, TREE-PUZZLE 5.2 [189,190] was run, using alignments created in ClustalW2, to generate the parameters such as the alpha (Gamma rate heterogeneity parameter) values, number of rate categories, individual rates and their respective probabilities to specify, as appropriate, coefficient of variation for a Gamma distribution model and how many categories of substitution rate there would be in a HMM of rate variation.

Protein trees were displayed using the TreeView visualisation tool:



### Genevestigator

We profiled the expression of senescence-related genes in *Arabidopsis *using the Genevestigator reference expression database and meta-analysis system for Affymetrix^® ^expression arrays [191]. Figure 2 presents output in heat-map format from the Meta-profile analysis tool based on screening of the ATH1 22 k array source for strength of expression categorised by Anatomy. Use of the Clustering analysis tool is shown in Figure 20. Both Anatomy and Gene categories were subjected to Hierarchical Clustering. Genevestigator displays absolute values either as MAS5-normalized signal values, or as MAS5 normalized values relative to the expression potential of each gene. The MAS5 normalization algorithm implemented by Genevestigator is described by Gentleman et al. [192]. The expression potential of a given gene is defined as the average of the top 1% signal values of a probe set across all arrays in the database. Its value is represented by the darkest blue colour, against which other levels of expression are normalised.



## Competing interests

The authors declare that they have no competing interests.

## Authors' contributions

HT conceived of the study, participated in sequence alignments and analyses of expression profiles and substantially drafted the manuscript. LH carried out the phylogeny and bootstrap analyses. MY was responsible for alignment and interpretation of Sgr-like sequences in *Clostridium*. HO devised the bioinformatics strategy, contributed to alignment and expression analyses and carried out the investigations of CCD8, NAC and cyanobacterial RCCR-like sequences. All authors read and approved the final manuscript.

## Supplementary Material

Additional file 1 **Outfiles from Consensus tree program, version 3.67**. Bootstrapping support values for protein consensus trees generated through the use of SEQBOOT, NEIGHBOR and CONSENSE and PUZZLEBOOT programs. Click here for file

Additional file 2 **Outfiles from PROML. Amino acid sequence Maximum Likelihood method, version 3.67**. Tree branch lengths with error estimates, generated using PROML with Gamma distribution. Click here for file

Additional file 3 **Synopsis of significant events in the evolution of plant senescence in relation to the geological timeline**. Innovations in the geological time-line relevant to the evolution of a senescence program integrated into plant development and adaptation. Click here for file

Additional file 4 **Log Likelihood (Ln) values corresponding to Hidden Markov Model, Gamma Distribution Model and Constant Rate Variation options in PHYLIP for phylogenetic analysis of senescence-related protein sequences**. Results of analysing all amino acid sequence data with the PROML program run with three different settings for the R (rate variation among sites) parameter. Click here for file
