# Supplementary material for: Evolution of plant senescence
Source: BMC Evol Biol. 2009 Jul 14;9:163. doi: 10.1186/1471-2148-9-163 (PMC2716323; doi:10.1186/1471-2148-9-163)
Supplement: Additional file 1 — Outfiles from Consensus tree program, version 3.67. Bootstrapping support values for protein consensus trees generated through the use of SEQBOOT, NEIGHBOR and CONSENSE and PUZZLEBOOT programs. [file 1471-2148-9-163-S1.doc]

**Additional File 3: Outfiles from Consensus tree program, version 3.67**

All trees are unrooted.

(a) RCCR (Figure 2)

Species in order:

1. Populus

2. Physcomitrella

3. Picea

4. Nostoc punctiforme

5. Anabaena

6. Nostoc sp

7. Marchantia

8. Pinus

9. Oryza

10. Festuca

11. Arabidopsis

Sets included in the consensus tree

Set (species in order) How many times out of 100.00

...***.... . 100.00

.********* . 100.00

....**.... . 100.00

........** . 100.00

.**...**.. . 81.00

.*******.. . 68.00

.*....*... . 56.00

.*....**.. . 53.00

Sets NOT included in consensus tree:

Set (species in order) How many times out of 100.00

.**...*... . 25.00

...***..** . 20.00

......**.. . 18.00

.**....... . 16.00

.**....*.. . 14.00

...****... . 12.00

.**...**** . 12.00

.*.....*.. . 10.00

.******... . 4.00

...*****.. . 3.00

..*....*.. . 2.00

.*.*****.. . 2.00

...***.*.. . 2.00

..****.... . 1.00

.*****.... . 1.00

Extended majority rule consensus tree

CONSENSUS TREE:

the numbers on the branches indicate the number

of times the partition of the species into the two sets

which are separated by that branch occurred

among the trees, out of 100.00 trees

+------Marchantia

+-56.0-|

+-53.0-| +------Physcomitrella

| |

+-81.0-| +-------------Pinus

| |

| +--------------------Picea

+-68.0-|

| | +------Anabaena

| | +100.0-|

| +-------100.0-| +------Nostoc sp

+100.0-| |

| | +-------------Nostoc punctiforme

| |

+------| | +------Oryza

| | +---------------------100.0-|

| | +------Festuca

| |

| +-----------------------------------------Arabidopsis

|

+------------------------------------------------Populus

_________________________________________________________________________

(b) PaO (Figure 3)

Species in order:

1. Vitis

2. Zea

3. Oryza

4. Phenylobacterium

5. Ostreococcus

6. Ralstonia

7. Chlamydomonas

8. Lyngbya

9. Cyanothece

10. Picea

11. Physcomitrella

12. Arabidopsis

Sets included in the consensus tree

Set (species in order) How many times out of 100.00

.......**. .. 100.00

...******. .. 100.00

.........* *. 100.00

.**....... .. 100.00

...****... .. 99.00

...*.*.... .. 65.00

...***.... .. 61.00

.********. .. 58.00

.........* ** 50.00

Sets NOT included in consensus tree:

Set (species in order) How many times out of 100.00

.********* *. 31.00

...******* *. 30.00

...******* ** 26.00

....**.... .. 19.00

...*..*... .. 18.00

...*.**... .. 17.00

...**..... .. 9.00

....***... .. 3.00

....*.*... .. 3.00

...**.*... .. 3.00

.....**... .. 2.00

...******. .* 2.00

.**....... .* 1.00

.********. .* 1.00

...***.**. .. 1.00

.**......* ** 1.00

Extended majority rule consensus tree

CONSENSUS TREE:

the numbers on the branches indicate the number

of times the partition of the species into the two sets

which are separated by that branch occurred

among the trees, out of 100.00 trees

+-------------Arabidopsis

+----------------------50.0-|

| | +------Physcomitrella

| +100.0-|

| +------Picea

|

| +------Lyngbya

+------| +--------------100.0-|

| | | +------Cyanothece

| | +100.0-|

| | | | +--------------------Chlamydomonas

| | | +-99.0-|

| | | | +-------------Ostreococcus

| | | +-61.0-|

| +-58.0-| | +------Ralstonia

| | +-65.0-|

| | +------Phenylobacterium

| |

| | +------Zea

| +---------------------100.0-|

| +------Oryza

|

+------------------------------------------------Vitis

_________________________________________________________________________

(c) Sgr (Figure 5)

Species in order:

1. Lycopersicon

2. Bacillus

3. Clostridium

4. Ostreococcus

5. Chlamydomonas

6. Physcomitrella

7. Zea

8. Oryza

9. Picea

10. Arabidopsi

Sets included in the consensus tree

Set (species in order) How many times out of 100.00

.********. 100.00

.****..... 100.00

......**.. 100.00

...**..... 99.00

.**....... 85.00

.....*..*. 67.00

.*****..*. 63.00

Sets NOT included in consensus tree:

Set (species in order) How many times out of 100.00

.*****.... 33.00

.****.**.. 19.00

......***. 9.00

.....****. 8.00

.*.**..... 8.00

..***..... 7.00

.*******.. 1.00

.**.*..... 1.00

Extended majority rule consensus tree

CONSENSUS TREE:

the numbers on the branches indicate the number

of times the partition of the species into the two sets

which are separated by that branch occurred

among the trees, out of 100.00 trees

+------Physcomitrella

+--------67.0-|

| +------Picea

|

+-63.0-| +------Clostridium

| | +-85.0-|

| | | +------Bacillus

| +100.0-|

+100.0-| | +------Ostreococcus

| | +-99.0-|

| | +------Chlamydomonas

| |

+------| | +------Oryza

| | +--------------100.0-|

| | +------Zea

| |

| +----------------------------------Arabidopsis

|

+-----------------------------------------Lycopersicon

_________________________________________________________________________

(d) WBC23 (Figure 7)

Species in order:

1. Vitis

2. Zea

3. Oryza

4. Ostreococcus

5. Monosiga

6. Nematostella

7. Chlamydomonas

8. Physcomitrella

9. Arabidopsis

Sets included in the consensus tree

Set (species in order) How many times out of 100.00

...****.. 100.00

...*****. 100.00

.**...... 100.00

...***... 99.00

....**... 96.00

.*******. 89.00

Sets NOT included in consensus tree:

Set (species in order) How many times out of 100.00

.**.....* 9.00

...**.... 4.00

...****** 2.00

....***.. 1.00

Extended majority rule consensus tree

CONSENSUS TREE:

the numbers on the branches indicate the number

of times the partition of the species into the two sets

which are separated by that branch occurred

among the trees, out of 100.00 trees

+---------------------------Physcomitrella

|

| +------Nematostella

+100.0-| +-96.0-|

| | +-99.0-| +------Monosiga

| | | |

| +100.0-| +-------------Ostreococcus

+-89.0-| |

| | +--------------------Chlamydomonas

| |

+------| | +------Zea

| | +---------------------100.0-|

| | +------Oryza

| |

| +-----------------------------------------Arabidopsis

|

+------------------------------------------------Vitis

_________________________________________________________________________

(e) Wrky53 (Figure 8)

Species in order:

1. Vitis

2. Chlamydomonas

3. Oryza

4. Hordeum

5. Physcomitrella

6. Arabidopsis

Sets included in the consensus tree

Set (species in order) How many times out of 100.00

..**.. 87.00

.****. 59.00

..***. 51.00

Sets NOT included in consensus tree:

Set (species in order) How many times out of 100.00

.***.. 40.00

....** 21.00

.*...* 13.00

...**. 7.00

.***.* 6.00

..**** 5.00

..*.*. 4.00

.*..*. 3.00

.*..** 2.00

.**.*. 1.00

.*.*.. 1.00

Extended majority rule consensus tree

CONSENSUS TREE:

the numbers on the branches indicate the number

of times the partition of the species into the two sets

which are separated by that branch occurred

among the trees, out of 100.00 trees

+------Oryza

+-87.0-|

+-51.0-| +------Hordeum

| |

+-59.0-| +-------------Physcomitrella

| |

+------| +--------------------Chlamydomonas

| |

| +---------------------------Arabidopsis

|

+----------------------------------Vitis

_________________________________________________________________________

(f) AtNAP (Figure 9)

Species in order:

1. Vitis

2. Oryza

3. Triticum

4. Picea

5. Physcomitrella

6. Arabidopsis

Sets included in the consensus tree

Set (species in order) How many times out of 100.00

.****. 100.00

.**... 100.00

...**. 100.00

Sets NOT included in consensus tree: NONE

Extended majority rule consensus tree

CONSENSUS TREE:

the numbers on the branches indicate the number

of times the partition of the species into the two sets

which are separated by that branch occurred

among the trees, out of 100.00 trees

+------Oryza

+100.0-|

| +------Triticum

+100.0-|

| | +------Physcomitrella

+------| +100.0-|

| | +------Picea

| |

| +--------------------Arabidopsis

|

+---------------------------Vitis

_________________________________________________________________________

(g) dee4 (Figure 10)

Species in order:

1. Physcomitrella

2. Vitis

3. Oryza

4. Zea

5. Auxenochlorella

6. Arabidopsis

7. Picea

Sets included in the consensus tree

Set (species in order) How many times out of 100.00

..**... 96.00

.****.. 94.00

..***.. 74.00

.....** 72.00

Sets NOT included in consensus tree:

Set (species in order) How many times out of 100.00

.****.* 19.00

.*..*.. 16.00

.***... 9.00

.*****. 5.00

.***..* 4.00

....**. 4.00

...**.. 2.00

..*.*.. 2.00

.***.** 2.00

..**..* 1.00

Extended majority rule consensus tree

CONSENSUS TREE:

the numbers on the branches indicate the number

of times the partition of the species into the two sets

which are separated by that branch occurred

among the trees, out of 100.00 trees

+------Picea

+---------------72.0-|

| +------Arabidopsis

|

+------| +-------------Auxenochlorella

| | +-74.0-|

| | | | +------Oryza

| +-94.0-| +-96.0-|

| | +------Zea

| |

| +--------------------Vitis

|

+----------------------------------Physcomitrella

_________________________________________________________________________

(h) PAP-Fibrillin (Figure 12)

Species in order:

1. Zea

2. Oryza

3. Picea

4. Arabidopsis

5. Vitis

6. Cyanothece

7. Nostoc

8. Ostreococcus

9. Chlamydomonas

10. Physcomitrella

Sets included in the consensus tree

Set (species in order) How many times out of 100.00

.....**... 100.00

..******** 100.00

.....***** 97.00

.....****. 93.00

...**..... 87.00

.......**. 59.00

..***..... 44.00

Sets NOT included in consensus tree:

Set (species in order) How many times out of 100.00

.....***.. 31.00

..*..***** 26.00

...******* 23.00

.....**.*. 10.00

...*.***** 9.00

..**.***** 8.00

.......*.* 3.00

..**...... 2.00

..***....* 1.00

.....***.* 1.00

.....**..* 1.00

...******. 1.00

...**..*.. 1.00

.......*** 1.00

.....**.** 1.00

....*..*.. 1.00

Extended majority rule consensus tree

CONSENSUS TREE:

the numbers on the branches indicate the number

of times the partition of the species into the two sets

which are separated by that branch occurred

among the trees, out of 100.00 trees

+------Nostoc

+100.0-|

| +------Cyanothece

+-93.0-|

| | +------Ostreococcus

+-97.0-| +-59.0-|

| | +------Chlamydomonas

| |

+100.0-| +--------------------Physcomitrella

| |

| | +------Arabidopsis

| | +-87.0-|

+------| +--------44.0-| +------Vitis

| | |

| | +-------------Picea

| |

| +----------------------------------Oryza

|

+-----------------------------------------Zea

_________________________________________________________________________

(i) CCD8 (Figure 13)

Species in order:

1. Oryza

2. Vitis

3. Zea

4. Ostreococcus

5. Hordeum

6. Cyanothece

7. Synechococcus

8. Picea

9. Coxiella

10. Chlamydomonas

11. Arabidopsis

12. Taxodium

13. Physcomitrella

Sets included in the consensus tree

Set (species in order) How many times out of 100.00

.....***.. ... 100.00

..***..... ... 100.00

.....**... ... 100.00

..******** ... 100.00

.......... **. 100.00

..******.. ... 100.00

...**..... ... 100.00

..******** *** 99.00

..******** ..* 86.00

..*******. ... 77.00

Sets NOT included in consensus tree:

Set (species in order) How many times out of 100.00

..******.* ... 17.00

..******** **. 14.00

........** ... 6.00

.*........ **. 1.00

Extended majority rule consensus tree

CONSENSUS TREE:

the numbers on the branches indicate the number

of times the partition of the species into the two sets

which are separated by that branch occurred

among the trees, out of 100.00 trees

+-------------Picea

+100.0-|

| | +------Synechococcus

| +100.0-|

+100.0-| +------Cyanothece

| |

| | +-------------Zea

| +100.0-|

+-77.0-| | +------Hordeum

| | +100.0-|

| | +------Ostreococcus

+100.0-| |

| | +---------------------------Coxiella

+-86.0-| |

| | +----------------------------------Chlamydomonas

| |

+-99.0-| +-----------------------------------------Physcomitrella

| |

| | +------Arabidopsis

+------| +-----------------------------------100.0-|

| | +------Taxodium

| |

| +-------------------------------------------------------Vitis

|

+--------------------------------------------------------------Oryza

_________________________________________________________________________

(j) OrI (Figure 14a)

Species in order:

1. Oryza

2. Physcomitrella

3. Ostreococcus

4. Chlamydomonas

5. Picea

6. Vitis

7. Brassica

Sets included in the consensus tree

Set (species in order) How many times out of 100.00

..**... 100.00

.****.. 99.00

.*****. 87.00

.***... 78.00

Sets NOT included in consensus tree:

Set (species in order) How many times out of 100.00

.*..*.. 22.00

.****.* 7.00

.....** 6.00

....**. 1.00

Extended majority rule consensus tree

CONSENSUS TREE:

the numbers on the branches indicate the number

of times the partition of the species into the two sets

which are separated by that branch occurred

among the trees, out of 100.00 trees

+----------------------------------Brassica

|

| +-------------Physcomitrella

+------| +-78.0-|

| | | | +------Chlamydomonas

| | +-99.0-| +100.0-|

| | | | +------Ostreococcus

| +-87.0-| |

| | +--------------------Picea

| |

| +---------------------------Vitis

|

+-----------------------------------------Oryza

_________________________________________________________________________

(k) OrII (Figure 14b)

Species in order:

1. Vitis

2. Oryza

3. Picea

4. Ostreococcus

5. Chlamydomonas

6. Physcomitrella

7. Arabidopsis

Sets included in the consensus tree

Set (species in order) How many times out of 100.00

...**.. 97.00

..****. 82.00

...***. 79.00

.*****. 64.00

Sets NOT included in consensus tree:

Set (species in order) How many times out of 100.00

.*....* 21.00

..*..*. 20.00

.*.**** 10.00

.*.***. 6.00

...**.* 4.00

..***** 3.00

...**** 3.00

.*.**.. 2.00

.**..*. 2.00

..**.*. 1.00

.**...* 1.00

.*.**.* 1.00

.***.*. 1.00

....**. 1.00

.**.... 1.00

...*.*. 1.00

Extended majority rule consensus tree

CONSENSUS TREE:

the numbers on the branches indicate the number

of times the partition of the species into the two sets

which are separated by that branch occurred

among the trees, out of 100.00 trees

+-------------Physcomitrella

+-79.0-|

| | +------Chlamydomonas

+-82.0-| +-97.0-|

| | +------Ostreococcus

+-64.0-| |

| | +--------------------Picea

+------| |

| | +---------------------------Oryza

| |

| +----------------------------------Arabidopsis

|

+-----------------------------------------Vitis

_________________________________________________________________________

(l) Bronze 1 (Figure 15)

Species in order:

1. Hordeum

2. Arabidopsis

3. Vitis

4. Picea

5. Physcomitrella

6. Chlamydomonas

7. Zea

Sets included in the consensus tree

Set (species in order) How many times out of 100.00

.*****. 100.00

.**.... 100.00

...**.. 99.00

...***. 99.00

Sets NOT included in consensus tree:

Set (species in order) How many times out of 100.00

...*.*. 1.00

.**..*. 1.00

Extended majority rule consensus tree

CONSENSUS TREE:

the numbers on the branches indicate the number

of times the partition of the species into the two sets

which are separated by that branch occurred

among the trees, out of 100.00 trees

+------Arabidopsis

+-------100.0-|

| +------Vitis

+100.0-|

| | +------Physcomitrella

| | +-99.0-|

+------| +-99.0-| +------Picea

| | |

| | +-------------Chlamydomonas

| |

| +---------------------------Zea

|

+----------------------------------Hordeum

_________________________________________________________________________

(m) C1 (Figure 16)

Species in order:

1. Oryza

2. Pinus

3. Physcomitrella

4. Ginkgo

5. Arabidopsis

6. Ostreococcus

7. Chlamydomonas

8. Picea

9. Vitis

10. Zea

Sets included in the consensus tree

Set (species in order) How many times out of 100.00

.....**... 100.00

.********. 100.00

.......**. 100.00

...**..... 94.00

.****..... 62.00

.****..**. 52.00

..***..... 40.00

Sets NOT included in consensus tree:

Set (species in order) How many times out of 100.00

.******... 42.00

.**....... 23.00

.*.**..... 18.00

..*....**. 15.00

.*...**... 13.00

.*.****... 13.00

...****... 10.00

..***..**. 8.00

..**...... 3.00

....***... 2.00

.**....**. 1.00

.*.*...... 1.00

.*..***... 1.00

.*..*..... 1.00

..*******. 1.00

Extended majority rule consensus tree

CONSENSUS TREE:

the numbers on the branches indicate the number

of times the partition of the species into the two sets

which are separated by that branch occurred

among the trees, out of 100.00 trees

+------Chlamydomonas

+---------------------100.0-|

| +------Ostreococcus

|

| +-------------Physcomitrella

+100.0-| +-40.0-|

| | | | +------Arabidopsis

| | +-62.0-| +-94.0-|

| | | | +------Ginkgo

| | | |

+------| +-52.0-| +--------------------Pinus

| | |

| | | +------Picea

| | +--------------100.0-|

| | +------Vitis

| |

| +-----------------------------------------Zea

|

+------------------------------------------------Oryza

_________________________________________________________________________

(n) AtMRP2 (Figure 17)

Species in order:

1. Physcomitrella

2. Cyanothece

3. Bdellovibrio

4. Chlamydomonas

5. Ostreococcus

6. Zea

7. Vitis

8. Oryza

9. Arabidopsis

Sets included in the consensus tree

Set (species in order) How many times out of 100.00

.*****... 100.00

...**.... 100.00

......*** 100.00

.......** 92.00

.**...... 75.00

.****.... 50.00

Sets NOT included in consensus tree:

Set (species in order) How many times out of 100.00

.**..*... 27.00

.*...*... 13.00

.*.***... 11.00

.*.**.... 10.00

...***... 10.00

......*.* 8.00

..*..*... 4.00

Extended majority rule consensus tree

CONSENSUS TREE:

the numbers on the branches indicate the number

of times the partition of the species into the two sets

which are separated by that branch occurred

among the trees, out of 100.00 trees

+------Chlamydomonas

+100.0-|

| +------Ostreococcus

+-50.0-|

| | +------Cyanothece

+100.0-| +-75.0-|

| | +------Bdellovibrio

| |

+------| +--------------------Zea

| |

| | +-------------Vitis

| +-------100.0-|

| | +------Oryza

| +-92.0-|

| +------Arabidopsis

|

+----------------------------------Physcomitrella

_________________________________________________________________________

(o) SEE2 (Figure 18)

Species in order:

1. Volvox

2. Ostreococcus

3. Physcomitrella

4. Picea

5. Lycopersicon

6. Arabidopsis

7. Oryza

8. Zea

9. Chlamydomonas

Sets included in the consensus tree

Set (species in order) How many times out of 100.00

......**. 98.00

..******. 97.00

....**... 87.00

....****. 85.00

..******* 79.00

...*****. 71.00

Sets NOT included in consensus tree:

Set (species in order) How many times out of 100.00

.*******. 21.00

..**..... 18.00

....*.**. 12.00

..*.****. 7.00

..****... 3.00

.*..****. 3.00

..***.**. 3.00

.*.*****. 3.00

.*....**. 3.00

...*..**. 3.00

..**..**. 2.00

...***... 2.00

.*....*.. 2.00

..*...**. 1.00

Extended majority rule consensus tree

CONSENSUS TREE:

the numbers on the branches indicate the number

of times the partition of the species into the two sets

which are separated by that branch occurred

among the trees, out of 100.00 trees

+------Arabidopsis

+-87.0-|

| +------Lycopersicon

+-85.0-|

| | +------Zea

+-71.0-| +-98.0-|

| | +------Oryza

+-97.0-| |

| | +--------------------Picea

+-79.0-| |

| | +---------------------------Physcomitrella

+------| |

| | +----------------------------------Chlamydomonas

| |

| +-----------------------------------------Ostreococcus

|

+------------------------------------------------Volvox

_________________________________________________________________________
