# Supplementary material for: Evolution of plant senescence
Source: BMC Evol Biol. 2009 Jul 14;9:163. doi: 10.1186/1471-2148-9-163 (PMC2716323; doi:10.1186/1471-2148-9-163)
Supplement: Additional file 2 — Outfiles from PROML. Amino acid sequence Maximum Likelihood method, version 3.67. Tree branch lengths with error estimates, generated using PROML with Gamma distribution. [file 1471-2148-9-163-S2.doc]

**Additional File 4: Outfiles from PROML. Amino acid sequence Maximum Likelihood method, version 3.67**

All analyses used Jones-Taylor-Thornton model of amino acid change. All trees are unrooted.

Branch lengths are given in amino acid substitutions per position.

(a) RCCR (Figure 2)

Discrete approximation to gamma distributed rates

Coefficient of variation of rates = 0.480000 (alpha = 4.340278)

States in HMM Rate of change Probability

1 0.265 0.066

2 0.606 0.341

3 1.075 0.404

4 1.689 0.164

5 2.474 0.025

6 3.471 0.0013

7 4.762 0.000018

8 6.560 0.000000

User-defined tree:

+Nostoc sp

+---5

+------------------------------------------4 +Anabaena

| |

| +--Nostoc punctiforme

|

+---3 +---------Marchantia

| | +--8

| | +--7 +-----Physcomitrella

| | | |

+---2 +--6 +-------------Pinus

| | |

| | +---Picea

| |

| | +----Festuca

| +----------9

| +--Oryza

|

1----Populus

|

+--------Arabidopsis

Ln Likelihood = -5238.43149

Between And Length Approx. Confidence Limits

------- --- ------ ------- ---------- ------

1 Arabidopsis 0.43846 ( 0.31960, 0.55733) **

1 2 0.19966 ( 0.09487, 0.30449) **

2 3 0.21853 ( 0.09149, 0.34514) **

3 4 2.13829 ( 1.67038, 2.60616) **

4 5 0.18963 ( 0.10795, 0.27139) **

5 Nostoc 0.02977 ( 0.00476, 0.05479) **

5 Anabaena 0.02147 ( zero, 0.04388) **

4 Nostoc punctiforme0.12786 ( 0.04924, 0.20650) **

3 6 0.15555 ( 0.02906, 0.28205) **

6 7 0.15438 ( 0.06319, 0.24555) **

7 8 0.08505 ( zero, 0.17631) *

8 Marchantia 0.52639 ( 0.36452, 0.68823) **

8 Physcomitrella 0.32116 ( 0.20943, 0.43287) **

7 Pinus 0.69692 ( 0.53558, 0.85825) **

6 Picea 0.19500 ( 0.10845, 0.28156) **

2 9 0.56522 ( 0.41688, 0.71357) **

9 Festuca 0.23775 ( 0.16240, 0.31310) **

9 Oryza 0.15148 ( 0.08377, 0.21920) **

1 Populus 0.27433 ( 0.17558, 0.37306) **

* = significantly positive, P < 0.05

** = significantly positive, P < 0.01

Combination of categories that contributes the most to the likelihood:

2333433333 3333323333 3333333233 3332323333 3333333333 3333433333

3333334324 2333323433 3332422323 2323432322 3333232342 2232132222

2443232222 3322322233 3232232223 3332333222 2133322222 2332323321

2321212233 2222333223 2332442243 3232332223 2222321223 1212333233

3332343433 3333332333 3433232233 3223334343 3333324242 3442431134

2233321313 2242322222 3323222232 2333433332

Most probable category at each site if > 0.95 probability ("." otherwise)

.......... .......... .......... .......... .......... ..........

.......... .......... .......... .......... .......... ..........

.......... .......... .......... .......... .......... ..........

.......... .......... .......... .......... .......... ..........

.......... .......... .......... .......... .......... ..........

.......... .......... .......... ..........

_________________________________________________________________________

(b) PaO (Figure 3)

Discrete approximation to gamma distributed rates

Coefficient of variation of rates = 0.680000 (alpha = 2.162630)

States in HMM Rate of change Probability

1 0.210 0.171

2 0.678 0.434

3 1.421 0.304

4 2.465 0.082

5 3.857 0.0089

6 5.679 0.00036

7 8.088 0.000004

8 11.496 0.000000

User-defined tree:

+-Vitis

|

| +---Cyanothece

| +------1

| | +--Lyngbya

+--8 |

| | +-------2 +--------------Ostreococcus

| | | | +--4

| | | | | | +------------------------Ralstonia

| | | +--3 +-------5

| +--6 | +-------------------------------Phenylobacterium

| | |

| | +----------Chlamydomonas

| |

| | +Oryza

| +--7

| +Zea

|

| +-Picea

9-10

| +--Physcomitrella

|

+-Arabidopsis

Ln Likelihood = -10617.78349

Between And Length Approx. Confidence Limits

------- --- ------ ------- ---------- ------

9 Arabidopsis 0.22396 ( 0.16618, 0.28174) **

9 8 0.08942 ( 0.04619, 0.13266) **

8 Vitis 0.15694 ( 0.10929, 0.20458) **

8 6 0.09971 ( 0.04509, 0.15433) **

6 2 0.77884 ( 0.57688, 0.98080) **

2 1 0.73564 ( 0.52947, 0.94181) **

1 Cyanothece 0.35886 ( 0.25590, 0.46181) **

1 Lyngbya 0.30345 ( 0.20508, 0.40180) **

2 3 0.33513 ( 0.14146, 0.52879) **

3 4 0.07655 ( zero, 0.34695)

4 Ostreococcus 1.47720 ( 1.16310, 1.79129) **

4 5 0.76594 ( 0.26273, 1.26915) **

5 Ralstonia 2.48285 ( 1.79440, 3.17128) **

5 Phenylobacterium 3.17428 ( 2.35684, 3.99170) **

3 Chlamydomonas 1.05863 ( 0.81040, 1.30686) **

6 7 0.13200 ( 0.07681, 0.18717) **

7 Oryza 0.05609 ( 0.02528, 0.08691) **

7 Zea 0.06201 ( 0.03335, 0.09067) **

9 10 0.18880 ( 0.12508, 0.25254) **

10 Picea 0.23399 ( 0.16850, 0.29946) **

10 Physcomitrella 0.27724 ( 0.20833, 0.34614) **

* = significantly positive, P < 0.05

** = significantly positive, P < 0.01

Combination of categories that contributes the most to the likelihood:

2222222222 2222222222 2222223322 3322222222 2222222222 3233332322

3332222222 2222222222 2222222222 2222222222 2222222343 3333433342

3433333333 3232334323 2332333323 3333333322 2222222222 2222222223

3334333434 4424333332 2422321111 2321122233 1222211132 2122223322

3231231211 1121111211 2222222141 2121111212 3312113111 2332222223

2332212113 3211322211 1212212223 2222222222 2222231233 3213212332

3332222222 2222221123 2232112112 2121122111 1222121222 1222232233

2223322323 2333223232 2232222332 2233333222 3232323232 2222212312

2212222222 2222223332 2232233221 2212321322 2131222121 2122112323

3233332232 2233223222 2122112213 1212223222 2232323333 3333322122

3222221112 2111331211 2312332233 2242333222 3322222232 4322222223

3233323412 3222322222 3233222222 2322223221 2212223222 2222222222

2222222222 2222222222 2222222222 2222222222 2222222222 2222222222

2222222222 2222222222 2222222222 2222222222 2222222222 2222222222

2222222222 2222222222 2222222222 2222222222 2222222222 2222222222

2222222222 22222222

Most probable category at each site if > 0.95 probability ("." otherwise)

.......... .......... .......... .......... .......... ..........

.......... .......... .......... .......... .......... ..........

.......... .......... .......... .......... .......... ..........

.......... .......... .......... .......... 1......... ..........

......1... 11.1..1... .......... ....1..... ........1. ..........

.......... .......... ..1....... .......... .......... .....1....

.......... .......... ........1. .1..1....1 .......... ..........

.......... .......... .......... .......... .......... ..........

.......... .......... .......... .......... .......... ..........

.......... .......... ....11.... .......... .......... ..........

.......... .......... .......... .......... .......... ..........

.......... .......... .......... .......... .......... ..........

.......... .......... .......... .......... .......... ..........

.......... .......... .......... .......... .......... ..........

.......... .......... .......... .......... .......... ..........

.......... ........

_________________________________________________________________________

(c) Sgr (Figure 5)

Discrete approximation to gamma distributed rates

Coefficient of variation of rates = 1.010000 (alpha = 0.980296)

States in HMM Rate of change Probability

1 0.170 0.375

2 0.913 0.417

3 2.282 0.173

4 4.334 0.033

5 7.165 0.0027

6 10.948 0.000088

7 16.026 0.000001

8 23.288 0.000000

User-defined tree:

+--Lycopersicon

|

| +------------Chlamydomonas

| +----------5

| | +----------------Ostreococcus

| +------------------4

| | | +-------------------Bacillus

| | +---------------6

| +--3 +----------------Clostridium

| | |

| | | +-----Picea

1---2 +--7

| | +------Physcomitrella

| |

| | +-Zea

| +---8

| +-Oryza

|

+--Arabidopsis

Ln Likelihood = -5440.12308

Between And Length Approx. Confidence Limits

------- --- ------ ------- ---------- ------

1 Arabidopsis 0.29052 ( 0.17326, 0.40781) **

1 Lycopersicon 0.25932 ( 0.14738, 0.37127) **

1 2 0.30797 ( 0.16312, 0.45281) **

2 3 0.00010 ( zero, 0.13166)

3 4 1.58299 ( 0.92372, 2.24227) **

4 5 0.94927 ( 0.35300, 1.54553) **

5 Chlamydomonas 1.04800 ( 0.56512, 1.53089) **

5 Ostreococcus 1.43513 ( 0.89107, 1.97919) **

4 6 1.35095 ( 0.60454, 2.09736) **

6 Bacillus 1.67540 ( 0.95662, 2.39420) **

6 Clostridium 1.38612 ( 0.71806, 2.05419) **

3 7 0.25575 ( 0.09275, 0.41875) **

7 Picea 0.53947 ( 0.35032, 0.72861) **

7 Physcomitrella 0.59103 ( 0.38429, 0.79777) **

2 8 0.32738 ( 0.18570, 0.46909) **

8 Zea 0.19208 ( 0.11111, 0.27307) **

8 Oryza 0.12619 ( 0.05293, 0.19943) **

* = significantly positive, P < 0.05

** = significantly positive, P < 0.01

Combination of categories that contributes the most to the likelihood:

2222222222 2222222222 2222222232 2323223222 3331223322 2322332333

2322232223 2221231222 1111111122 1211112121 2222213232 2223221211

1111111211 1111122112 2111221111 2111111112 2121221111 1211111212

2222221321 2211111111 1121111112 2113221112 2121212111 2222222222

2222222222 2222222222 2222222222 2222222222 2222222222 2222222222

2222223211 2222222222 2222222222 2222222222 2222222222 2222222222

2222222221 2222132222 3233333332 3233332332 3323222222 2222222222

2222222222 2323222211 2223222222 2332322223 3222222232 2222212222

2222222222 2222222222 2222222222 2222222222 2222222222 2222222222

2222222222 2222222222 2222222222 2222222222 2222222222 2222222222

2222222222 2222222222 2222222222 2222222222 2222222222 2222222222

2222222222 2222222222 2222222222 2222222222 2222222222 2222222222

2222222222 2222222222 2222222222 222222

Most probable category at each site if > 0.95 probability ("." otherwise)

.......... .......... .......... .......... .......... ..........

.......... .......... ...1...... 1.1.11.1.. .......... ......1.11

11111.1..1 .1.11...1. .1........ .1111.1... ........11 1..1111...

.........1 ..11.11111 11.1...11. .1....1.1. .1.1.1.1.1 ..........

.......... .......... .......... .......... .......... ..........

.......... .......... .......... .......... .......... ..........

.........1 .......... .......... .......... .......... ..........

.......... .......... .......... .......... .......... ..........

.......... .......... .......... .......... .......... ..........

.......... .......... .......... .......... .......... ..........

.......... .......... .......... .......... .......... ..........

.......... .......... .......... .......... .......... ..........

.......... .......... .......... ......

_________________________________________________________________________

(d) WBC23 (Figure 7)

Discrete approximation to gamma distributed rates

Coefficient of variation of rates = 0.850000 (alpha = 1.384083)

States in HMM Rate of change Probability

1 0.184 0.277

2 0.783 0.442

3 1.821 0.227

4 3.337 0.049

5 5.401 0.0045

6 8.139 0.00016

7 11.794 0.000002

8 17.000 0.000000

User-defined tree:

+-Vitis

|

| +-Zea

| +--3

| | +Oryza

| |

| | +------------Nematostella

1--2 +-----7

| | +-----6 +----------------Monosiga

| | | |

| | +----5 +----------------------------Ostreococcus

| | | |

| +-----4 +-------------Chlamydomonas

| |

| +------Physcomitrella

|

+--Arabidopsis

Ln Likelihood = -10706.90131

Between And Length Approx. Confidence Limits

------- --- ------ ------- ---------- ------

1 Arabidopsis 0.16507 ( 0.12472, 0.20541) **

1 Vitis 0.12864 ( 0.09181, 0.16549) **

1 2 0.13012 ( 0.08033, 0.17989) **

2 3 0.18833 ( 0.13408, 0.24257) **

3 Zea 0.09334 ( 0.04671, 0.13997) **

3 Oryza 0.02402 ( zero, 0.05866)

2 4 0.36569 ( 0.26117, 0.47022) **

4 5 0.32926 ( 0.18800, 0.47051) **

5 6 0.38272 ( 0.19972, 0.56572) **

6 7 0.39193 ( 0.19902, 0.58485) **

7 Nematostella 0.82910 ( 0.63654, 1.02167) **

7 Monosiga 1.12812 ( 0.90686, 1.34937) **

6 Ostreococcus 1.91937 ( 1.56947, 2.26925) **

5 Chlamydomonas 0.96111 ( 0.76791, 1.15432) **

4 Physcomitrella 0.44637 ( 0.33777, 0.55497) **

* = significantly positive, P < 0.05

** = significantly positive, P < 0.01

Combination of categories that contributes the most to the likelihood:

2222233222 2333222122 3222232232 2232322332 2223233233 3323223222

3232112221 3233233232 2232223222 1222222212 2222222213 3323222222

2222222222 3122222223 2233232222 3322223322 2322223332 3233332231

2322222223 2133223322 2222113212 1213111111 2111111111 1112212113

3332333132 2212332221 3222222111 1212122211 1311122212 1111232312

2213321222 1321113222 2121122232 3111111111 1121121222 2112221111

1111111112 1222311221 3222111211 1111112222 3211121123 1322121212

3233213122 2312232111 1221122222 2222232221 3212223322 2222222233

3333332323 2323222221 2222232222 2222222211 2212332222 2233232332

2323331332 2323212231 2221231221 2112222213 2222123222 2112322222

2223322332 2123122112 2222222221 2122211212 2122111121 2111111221

1221111222 3213313321 1212112322 4312222221 2132212212 1121221222

2322121212 1222121222 2221232132 2212212121 2221221222 2233333232

2222221122 1222211233 2313233323 3332323321 3212323132 1321131113

2244322222 2222

Most probable category at each site if > 0.95 probability ("." otherwise)

.......... .......... .......... .......... .......... ..........

.......... .......... .......... .......... .......... ..........

.......... .......... .......... .......... .......... ..........

.......... .......... .......... ......1..1 .1..1...11 ........1.

.......... ..1....... ........1. 1........1 ..11...... .1........

.......... .......... .1........ .1..1..1.1 1..1..1... .......11.

11..11..1. .......... .....1.... .11..1.... ..1....... ..........

.......... .......1.. .......... .......... .......... ..........

.......... .......... .......... .......... .......... ..........

.......... .......... ......1... .......... .......... ..........

.......... .1........ .......... .1......1. .....11... ....1.....

.......... .......... .......... .......... .......... ......1...

........1. .......... .......... .......... .......... ..........

.......... .......... .......... .......... .......... ..........

.......... ....

_________________________________________________________________________

(e) Wrky53 (Figure 8)

Discrete approximation to gamma distributed rates

Coefficient of variation of rates = 0.300000 (alpha = 11.111111)

States in HMM Rate of change Probability

1 0.372 0.015

2 0.613 0.178

3 0.894 0.421

4 1.227 0.304

5 1.624 0.075

6 2.103 0.0061

7 2.701 0.00013

8 3.507 0.000000

User-defined tree:

+-------Vitis

|

| +---Oryza

| +-----4

| +--3 +----Hordeum

| | |

1----2 +--------------------------------------------------Physcomitrella

| |

| +--------------------------------Chlamydomonas

|

+--------Arabidopsis

Ln Likelihood = -5612.32423

Between And Length Approx. Confidence Limits

------- --- ------ ------- ---------- ------

1 Arabidopsis 0.57723 ( 0.41973, 0.73456) **

1 Vitis 0.52915 ( 0.37778, 0.68066) **

1 2 0.35594 ( 0.15663, 0.55264) **

2 3 0.16746 ( zero, 0.36936)

3 4 0.40720 ( 0.20356, 0.61084) **

4 Oryza 0.25566 ( 0.14174, 0.36960) **

4 Hordeum 0.32815 ( 0.21334, 0.44296) **

3 Physcomitrella 3.37000 ( 2.55557, 4.18444) **

2 Chlamydomonas 2.17026 ( 1.71537, 2.62515) **

* = significantly positive, P < 0.05

** = significantly positive, P < 0.01

Combination of categories that contributes the most to the likelihood:

3333333333 3333333333 3333333333 3333333333 3333333333 3333333333

3333333333 3333333333 3333333333 3333333333 3333333333 3333333343

3333433324 3333334333 3333343423 3343334434 3333343333 3343333333

3343333433 3333343233 3343433333 3333334443 3333333323 3233333233

3334333333 3333333443 3333344333 3333233333 3333432333 3343333333

3333333333 3333332333 3223322333 3333223332 3333222334 3333433223

3233333343 3333324233 3333333333 3333333333 3333333333 3333333333

3333333343 3334433333 3334334433 3343434333 4433343333 3343334333

4433433344 4434333333 3333334344 4344333434 4434444443 4443343333

3333333333 3333333333 3334433444 3333333333 3433333333 3333333

Most probable category at each site if > 0.95 probability ("." otherwise)

.......... .......... .......... .......... .......... ..........

.......... .......... .......... .......... .......... ..........

.......... .......... .......... .......... .......... ..........

.......... .......... .......... .......... .......... ..........

.......... .......... .......... .......... .......... ..........

.......... .......... .......... .......... .......... ..........

.......... .......... .......... .......... .......... ..........

.......... .......... .......... .......... .......... ..........

.......... .......... .......... .......... .......... ..........

.......... .......... .......... .......... .......... .......

_________________________________________________________________________

(f) AtNAP (Figure 9)

Discrete approximation to gamma distributed rates

Coefficient of variation of rates = 1.710000 (alpha = 0.341986)

States in HMM Rate of change Probability

1 0.142 0.673

2 1.777 0.252

3 5.254 0.066

4 10.717 0.0093

5 18.435 0.00064

6 28.895 0.000018

7 43.066 0.000000

8 63.464 0.000000

User-defined tree:

+------Vitis

|

| +Triticum

| +---------------------3

| | +Oryza

1------------2

| | +-------------Picea

| +----------------------4

| +---------Physcomitrella

|

+--------Arabidopsis

Ln Likelihood = -3028.34843

Between And Length Approx. Confidence Limits

------- --- ------ ------- ---------- ------

1 Arabidopsis 0.62086 ( 0.34593, 0.89509) **

1 Vitis 0.43349 ( 0.17080, 0.69641) **

1 2 0.87086 ( 0.32898, 1.41130) **

2 3 1.47383 ( 0.85415, 2.09221) **

3 Triticum 0.01469 ( zero, 0.03099) **

3 Oryza 0.00886 ( zero, 0.02481)

2 4 1.49184 ( 0.81721, 2.16653) **

4 Picea 0.91067 ( 0.41835, 1.40289) **

4 Physcomitrella 0.68065 ( 0.26292, 1.09839) **

* = significantly positive, P < 0.05

** = significantly positive, P < 0.01

Combination of categories that contributes the most to the likelihood:

1111111221 2122211221 1111111111 1111111121 1121111111 1111111111

1112111112 1111111111 1111111111 1111111111 1111111111 2122212221

1111111111 1111111111 1111111111 1111222111 1111111111 1111111123

1121112122 2211122221 1211222222 1212211122 2222222212 2222222211

2111211212 2221121122 2122221221 2111212222 1221222122 2211222112

2211111221 1111121111 1111111112 1111111212 2212211221 1222222222

2211121121

Most probable category at each site if > 0.95 probability ("." otherwise)

......1... .....1...1 1111111111 1111.111.. 1..1.1.111 1111111111

.11.11.11. 1111111111 111111111. 111111.111 1111111111 .1.......1

111111111. 1111111111 11111111.. ........11 111.111111 11111111..

.......... ....1..... .......... 1......... .......... ........1.

.11....... ....1.1... ......1... .......... .......... ..1.....1.

.......... .......... .......... .......... ......1..1 1.........

..........

_________________________________________________________________________

(g) dee4 (Figure 10)

Discrete approximation to gamma distributed rates

Coefficient of variation of rates = 0.910000 (alpha = 1.207584)

States in HMM Rate of change Probability

1 0.178 0.315

2 0.829 0.435

3 1.984 0.205

4 3.690 0.042

5 6.027 0.0037

6 9.136 0.00013

7 13.297 0.000001

8 19.232 0.000000

User-defined tree:

+---Physcomitrella

|

+--4 +---Vitis

| | |

| +--1 +Oryza

| | +--3

| +--2 +Zea

| |

| +-----------------------------------------------------Auxenochlorella

|

5--Arabidopsis

|

+----Picea

Ln Likelihood = -5824.43410

Between And Length Approx. Confidence Limits

------- --- ------ ------- ---------- ------

5 Picea 0.69250 ( 0.54938, 0.83567) **

5 4 0.10429 ( 0.02719, 0.18142) **

4 Physcomitrella 0.46839 ( 0.34893, 0.58789) **

4 1 0.35966 ( 0.23451, 0.48475) **

1 Vitis 0.51795 ( 0.38437, 0.65158) **

1 2 0.22481 ( 0.09642, 0.36682) **

2 3 0.29956 ( 0.16240, 0.43676) **

3 Oryza 0.06143 ( 0.01423, 0.10861) **

3 Zea 0.15375 ( 0.09683, 0.21067) **

2 Auxenochlorella 7.22447 ( 4.85681, 9.59213) **

5 Arabidopsis 0.40671 ( 0.29595, 0.51750) **

* = significantly positive, P < 0.05

** = significantly positive, P < 0.01

Combination of categories that contributes the most to the likelihood:

2222222323 3333312222 2222222222 2222222322 3233333223 2333221112

1221121111 1111112221 2111211221 1222332232 2311232122 1112212121

2221221221 2222113232 3212321222 2311322111 2222123122 2211223222

2223323322 2222222122 1221112122 2322231122 1121112112 1222223213

2223233231 2121222343 2322234223 2232222222 1211122212 3221111112

2111212221 2212322232 1131223223 3221121221 1112112212 3212212131

1322221121 1211221122 1321332122 2132322233 2322212222 2222222333

3332322232 1221132123 1131122311 1121212111 2212112112 1121222233

2212232233 1132122312 1132222112 3222222233 2213233

Most probable category at each site if > 0.95 probability ("." otherwise)

.......... .......... .......... .......... .......... ..........

.......... .......... .......... .......... .......... ..........

.......... .......... .......... .......... .......... ...1......

.......... .......... .....1.1.. .......1.. ....1..... ..........

.......... .......... .......... .......... .......... .......11.

.1........ .......... .......... .........1 .......... ..1.......

.......... .......... .......... .......... .......... ..........

.......... ...1...... 11......1. .........1 ....1..... 1.........

.......... .......... .1........ .......... .......

_________________________________________________________________________

(h) PAP-Fibrillin (Figure 12)

Discrete approximation to gamma distributed rates

Coefficient of variation of rates = 0.640000 (alpha = 2.441406)

States in HMM Rate of change Probability

1 0.218 0.147

2 0.659 0.424

3 1.341 0.324

4 2.288 0.093

5 3.543 0.011

6 5.178 0.00045

7 7.334 0.000005

8 10.377 0.000000

User-defined tree:

+---Arabidopsis

+--8

+--7 +-Vitis

| |

+--2 +--Picea

| |

| | +Oryza

| +--1

| +-Zea

|

| +---Nostoc

| +-----------------5

| | +-----Cyanothece

3--4

| | +---------------------------------------------Ostreococcus

| +----6

| +----------Chlamydomonas

|

+------Physcomitrella

Ln Likelihood = -6355.28840

Between And Length Approx. Confidence Limits

------- --- ------ ------- ---------- ------

3 Physcomitrella 0.60416 ( 0.45203, 0.75629) **

3 2 0.12257 ( 0.00970, 0.23542) **

2 7 0.09809 ( 0.02553, 0.17065) **

7 8 0.14106 ( 0.06791, 0.21422) **

8 Arabidopsis 0.35533 ( 0.26109, 0.44957) **

8 Vitis 0.19259 ( 0.11803, 0.26714) **

7 Picea 0.28177 ( 0.19371, 0.36982) **

2 1 0.28553 ( 0.18877, 0.38228) **

1 Oryza 0.08303 ( 0.03308, 0.13300) **

1 Zea 0.17187 ( 0.11090, 0.23283) **

3 4 0.22999 ( 0.01645, 0.44360) **

4 5 1.49137 ( 1.05308, 1.92963) **

5 Nostoc 0.35195 ( 0.17134, 0.53258) **

5 Cyanothece 0.47597 ( 0.28185, 0.67008) **

4 6 0.39235 ( 0.10952, 0.67510) **

6 Ostreococcus 3.86281 ( 2.83741, 4.88824) **

6 Chlamydomonas 0.92559 ( 0.62386, 1.22731) **

* = significantly positive, P < 0.05

** = significantly positive, P < 0.01

Combination of categories that contributes the most to the likelihood:

2222222222 2222222222 2222222222 2222222222 2222222222 2222222222

2222222222 2222222222 2222223222 2432333334 3423332323 3423333334

4332333432 3333333333 3334433333 3332323423 3233332223 4333344333

3323332222 3323233433 2234312313 2232122222 2221212131 2133122232

2122112312 2212222222 2222222322 2122222212 2231232223 2322222222

2222222222 2222223222 3212121222 2222222222 2333223122 2232313322

2312213222 2122222222 2222222221 2122222222 2222222222 2122222222

2223232223 2123222232 3222222222 2222322132 2232222222 2323232222

2222222222 2222222222 2222222222 2222222222 2222222222 2222222222

2222222222 2222222222 2222223222 1121112132 1111122221 2211312222

2242222222

Most probable category at each site if > 0.95 probability ("." otherwise)

.......... .......... .......... .......... .......... ..........

.......... .......... .......... .......... .......... ..........

.......... .......... .......... .......... .......... ..........

.......... .......... .......... .......... .......... ..........

.......... .......... .......... .......... .......... ..........

.......... .......... .......... .......... .......... ..........

.......... .......... .......... .......... .......... ..........

.......... .......... .......... .......... .......... ..........

.......... .......... .......... .......... .......... ..........

.......... .......... .......... ....1..... .......... ..........

..........

_________________________________________________________________________

(i) CCD8 (Figure 13)

Discrete approximation to gamma distributed rates

Coefficient of variation of rates = 0.640000 (alpha = 2.441406)

States in HMM Rate of change Probability

1 0.218 0.147

2 0.659 0.424

3 1.341 0.324

4 2.288 0.093

5 3.543 0.011

6 5.178 0.00045

7 7.334 0.000005

8 10.377 0.000000

User-defined tree:

+Taxodium

+--11

| +Arabidopsis

+--2

| | +-Vitis

| +--1

| +--Oryza

|

| +--------------Chlamydomonas

| |

| | +------Cyanothece

| | +-----8

| | +------7 +----Synechococcus

3--------4 | |

| | | +-------------------Picea

| | +------6

| | | | +-----Zea

| | | +-----------------9

| +--5 | +Hordeum

| | +--------10

| | +-Ostreococcus

| |

| +--------------Coxiella

|

+---Physcomitrella

Ln Likelihood = -15484.38216

Between And Length Approx. Confidence Limits

------- --- ------ ------- ---------- ------

3 Physcomitrella 0.35674 ( 0.26203, 0.45145) **

3 2 0.20464 ( 0.11865, 0.29062) **

2 11 0.30870 ( 0.23516, 0.38223) **

11 Taxodium 0.00010 ( zero, infinity)

11 Arabidopsis 0.00010 ( zero, infinity)

2 1 0.16097 ( 0.09924, 0.22268) **

1 Vitis 0.17978 ( 0.12617, 0.23339) **

1 Oryza 0.27296 ( 0.21045, 0.33546) **

3 4 0.72976 ( 0.52105, 0.93847) **

4 Chlamydomonas 1.25601 ( 1.00386, 1.50816) **

4 5 0.26544 ( 0.07944, 0.45144) **

5 6 0.60416 ( 0.34784, 0.86048) **

6 7 0.57449 ( 0.31078, 0.83820) **

7 8 0.48239 ( 0.27276, 0.69201) **

8 Cyanothece 0.61232 ( 0.47881, 0.74585) **

8 Synechococcus 0.36896 ( 0.25031, 0.48759) **

7 Picea 1.69427 ( 1.37224, 2.01628) **

6 9 1.47376 ( 1.12044, 1.82710) **

9 Zea 0.50908 ( 0.35387, 0.66429) **

9 10 0.78575 ( 0.61717, 0.95432) **

10 Hordeum 0.06701 ( 0.02722, 0.10677) **

10 Ostreococcus 0.15498 ( 0.10816, 0.20179) **

5 Coxiella 1.23612 ( 0.97084, 1.50138) **

* = significantly positive, P < 0.05

** = significantly positive, P < 0.01

Combination of categories that contributes the most to the likelihood:

2323332223 2333222222 3223322323 3233232233 3232223223 3333233333

2333443233 3333433324 3232234334 4333443323 3333323333 2334233333

3333333433 2232332243 3333412132 1332311221 1122232323 3322121111

2122223233 2222332221 2221222322 2332233222 2222232322 3232233333

2333323323 3212211222 3322222221 2212321233 2123321121 2122322231

4322222111 2322212333 2232232333 3323223222 2323223244 2223333313

4222221121 2121221221 3122212222 2322222232 2333233323 2323222222

3233332323 2222332211 2211212332 3323223122 1222222223 2332332332

2233222232 2222212312 2321322222 2222323233 2343332233 2122321233

3233221121 2221323223 2322221222 1312322322 2222222232 3322333332

3222113111 2222333323 1111222221 2213323122 2222232222 2132232332

1123213221 2121132223 3333333332 2223232221 2322232222 2223233222

2222232223 2222322213 2222222222 2222222222 2222222222 2222222222

2222222222 2222222222 2222222222 2222222222 2222222222 2222222222

2222222222 2222222222 2222222222 2222222222 2222222222 2222222222

2222222222 2222222222 2222222222 2222222222 2222222222 2222222222

2222222222 2222222222 2222222222 2222222222 2222222222 2222222222

2222222222 2222222222 2222222222 2222222222 2222222222 2222222222

2222222222 2222222222 2222222222 2222222222 2222222222 2222222222

2222222222 2222222222 2222222222 2222222222 2222222222 2222222222

2222222222 2222222222 2222222222 2222222222 2222222222 2222222222

2222222222 2222222222 2222222222 2222222222 2222222222 2222222222

2222222222 2222222222 2222222222 2222222222 2222222222 2222222222

2222222222 2222222222 2222222222 2222222222 2222222222 22222222

Most probable category at each site if > 0.95 probability ("." otherwise)

.......... .......... .......... .......... .......... ..........

.......... .......... .......... .......... .......... ..........

.......... .......... .......... .......... 1......... ....1..1..

.......... .......... ...1...... .......... .......... ..........

.......... ......1... .......... .......... ......1..1 ..........

.......11. .......... .......... .......... .......... ..........

......1... .1........ .......... .......... .......... ..........

.......... .........1 ..1....... .......... .......... ..........

.......... .....1.... .......... .......... .......... ......1...

.......... .......... .......... .......... .......... ..........

....1...1. .......... 1......... .......... .......... ..........

1......... ...1...... .......... .......... .......... ..........

.......... .......... .......... .......... .......... ..........

.......... .......... .......... .......... .......... ..........

.......... .......... .......... .......... .......... ..........

.......... .......... .......... .......... .......... ..........

.......... .......... .......... .......... .......... ..........

.......... .......... .......... .......... .......... ..........

.......... .......... .......... .......... .......... ..........

.......... .......... .......... .......... .......... ..........

.......... .......... .......... .......... .......... ..........

.......... .......... .......... .......... .......... ..........

.......... .......... .......... .......... .......... ..........

.......... .......... .......... .......... .......... ........

_________________________________________________________________________

(j) OrI (Figure 14a)

Discrete approximation to gamma distributed rates

Coefficient of variation of rates = 1.290000 (alpha = 0.600925)

States in HMM Rate of change Probability

1 0.154 0.520

2 1.202 0.348

3 3.286 0.112

4 6.493 0.018

5 10.978 0.0014

6 17.021 0.000040

7 25.175 0.000000

8 36.881 0.000000

User-defined tree:

+----Vitis

|

| +----Oryza

5--4

| | +----------------Picea

| +---1

| | +---Physcomitrella

| +--2

| | +------------------Chlamydomonas

| +--------3

| +--------------------------------Ostreococcus

|

+---Brassica

Ln Likelihood = -3626.05343

Between And Length Approx. Confidence Limits

------- --- ------ ------- ---------- ------

5 Brassica 0.19399 ( 0.11306, 0.27491) **

5 Vitis 0.22859 ( 0.14049, 0.31669) **

5 4 0.07567 ( 0.00744, 0.14389) **

4 Oryza 0.25830 ( 0.15569, 0.36089) **

4 1 0.20644 ( 0.08293, 0.32995) **

1 Picea 0.87610 ( 0.63697, 1.11523) **

1 2 0.08999 ( zero, 0.20033) *

2 Physcomitrella 0.18398 ( 0.06379, 0.30416) **

2 3 0.44324 ( 0.16824, 0.71824) **

3 Chlamydomonas 0.94228 ( 0.60550, 1.27908) **

3 Ostreococcus 1.67157 ( 1.17632, 2.16682) **

* = significantly positive, P < 0.05

** = significantly positive, P < 0.01

Combination of categories that contributes the most to the likelihood:

1323222212 2222223322 3232333232 3212233211 1122133222 3232222332

3221332232 2312222211 1111122223 2222221111 1121112111 1111211221

1211111111 1111111111 1111122222 2332222211 1122222121 1221212221

1131122222 2112112111 1211211111 1211111211 1212111211 1111111111

1111111111 1111121112 1112111211 2111111112 1121122222 1111223222

2222222122 1111111211 1111111111 1111111111 1111221111 111111131

Most probable category at each site if > 0.95 probability ("." otherwise)

.......... .......... .......... .......... .......... ..........

.......... .......... .......... .......111 1....1.111 .1...11..1

1.1111.111 .111.1111. 111....... .......... .1.....1.. 1..1.1....

.......... ....1..11. ..11..1..1 1.111.1..1 1.1.111.11 1111111111

.111111.1. ..111.111. .1....1.11 .11111.11. 1......... ..........

.......... 1..1.1..1. .......... ....1..... ..1...11.1 ....1....

_________________________________________________________________________

(k) OrII (Figure 14b)

Discrete approximation to gamma distributed rates

Coefficient of variation of rates = 1.680000 (alpha = 0.354308)

States in HMM Rate of change Probability

1 0.143 0.664

2 1.731 0.258

3 5.095 0.068

4 10.376 0.0097

5 17.833 0.00067

6 27.937 0.000019

7 41.622 0.000000

8 61.319 0.000000

User-defined tree:

+----Vitis

|

| +--Physcomitrella

| +---4

| | | +-----------------------Chlamydomonas

| +---3 +-----------5

| | | +-------------------------------Ostreococcus

1--2 |

| | +-------------Picea

| |

| +-------Oryza

|

+---Arabidopsis

Ln Likelihood = -3629.10309

Between And Length Approx. Confidence Limits

------- --- ------ ------- ---------- ------

1 Arabidopsis 0.25130 ( 0.12097, 0.38163) **

1 Vitis 0.32993 ( 0.18234, 0.47754) **

1 2 0.11761 ( zero, 0.24881) *

2 3 0.28276 ( 0.10047, 0.46504) **

3 4 0.23121 ( 0.04317, 0.41923) **

4 Physcomitrella 0.17132 ( 0.00956, 0.33309) **

4 5 0.79768 ( 0.32764, 1.26770) **

5 Chlamydomonas 1.59970 ( 1.00757, 2.19184) **

5 Ostreococcus 2.12830 ( 1.44524, 2.81137) **

3 Picea 0.92172 ( 0.60785, 1.23560) **

2 Oryza 0.52657 ( 0.33320, 0.71995) **

* = significantly positive, P < 0.05

** = significantly positive, P < 0.01

Combination of categories that contributes the most to the likelihood:

1111111111 1112221222 1122122221 2222122222 2121222222 2211222222

2221222222 2222232212 2212212211 1111111111 1111211211 1111111111

1211111111 1111111111 1111111112 2231122222 2122111112 1111111111

1111111111 1211211221 1111121111 1111111111 1111222211 2112111111

1111111111 1111111111 1111111111 1111111111 1111111121 1211111111

1111111111 1111211111 1111111111 1111111111 1121111111 1111111111

1111111111 1111111111 1111111111 1111111111 1111111111 1111111111

1111111111 1112111211 1211211111 2111112112 2222212232 2222221222

1121111111 1111111111 1111111111 1111111122 1111111111 1111111111

1111111111 1211111111 1111111111 1111111111 1111111111 1111111111

1111111111 1111111111 1111111111 1111111111 1111111111 1111111

Most probable category at each site if > 0.95 probability ("." otherwise)

.......... .......... ....1..... ....1..... .......... ..........

.......... .......... ........11 111111.111 .111.11..1 ......1..1

1.11111111 1...111111 11111111.. .......... ....11...2 11........

.........1 1..1..1..1 .1..1..... .......... ........1. .11.11....

.......... .......... .......... .......... ......11.1 1.11111111

1111.111.1 1111.11111 1111111111 1111111111 11.11..... ..........

.......... .......... .......... .......... .......... ..........

.......... ..1.111.11 1.11.11111 .11.11.11. .....1.... ..........

11..111111 1.1....... .......111 1111111... 11........ ..........

..11111.11 1......... .......... .......... .......... ..........

.......... .......... .......... .......... .......... .......

_________________________________________________________________________

(l) Bronze 1 (Figure 15)

Discrete approximation to gamma distributed rates

Coefficient of variation of rates = 0.450000 (alpha = 4.938272)

States in HMM Rate of change Probability

1 0.278 0.054

2 0.601 0.317

3 1.036 0.415

4 1.597 0.182

5 2.306 0.029

6 3.203 0.0016

7 4.359 0.000024

8 5.961 0.000000

User-defined tree:

+-Hordeum

|

| +----Arabidopsis

| +-----3

| | +---Vitis

1-----2

| | +------------Physcomitrella

| | +------5

| +------4 +---------Picea

| |

| +-------------------------------------------Chlamydomonas

|

+-Zea

Ln Likelihood = -6976.12733

Between And Length Approx. Confidence Limits

------- --- ------ ------- ---------- ------

1 Zea 0.14483 ( 0.09417, 0.19551) **

1 Hordeum 0.14656 ( 0.09550, 0.19762) **

1 2 0.41492 ( 0.29255, 0.53723) **

2 3 0.39864 ( 0.27591, 0.52137) **

3 Arabidopsis 0.34806 ( 0.26246, 0.43369) **

3 Vitis 0.28226 ( 0.20178, 0.36272) **

2 4 0.49105 ( 0.29174, 0.69277) **

4 5 0.42427 ( 0.22346, 0.62509) **

5 Physcomitrella 0.83186 ( 0.65603, 1.00771) **

5 Picea 0.65291 ( 0.49029, 0.81553) **

4 Chlamydomonas 2.94058 ( 2.32629, 3.55488) **

* = significantly positive, P < 0.05

** = significantly positive, P < 0.01

Combination of categories that contributes the most to the likelihood:

3333334333 3333222232 2232322233 2333232333 2333333322 3323433322

4323333333 4333223333 2322323324 3343233434 3233323333 3343233243

3333332333 2323333432 2313333333 2323222332 3333432243 2342333334

3333333324 3243342223 4432332323 3323323333 3333323333 4333323223

3222332233 3333223333 3333332233 2333333443 4333333332 3333323332

2322333333 2132222223 2332332333 3232223233 3222222333 3343333323

3233432333 2333333322 2222123223 1222322211 3232123122 2322223232

3222233123 3234233333 3333333223 3333323234 2332333334 3334333333

3333333223 3334233324 3224332333 3233333332 4343332333 3333333333

3333333333

Most probable category at each site if > 0.95 probability ("." otherwise)

.......... .......... .......... .......... .......... ..........

.......... .......... .......... .......... .......... ..........

.......... .......... .......... .......... .......... ..........

.......... .......... .......... .......... .......... ..........

.......... .......... .......... .......... .......... ..........

.......... .......... .......... .......... .......... ..........

.......... .......... .......... .......... .......... ..........

.......... .......... .......... .......... .......... ..........

.......... .......... .......... .......... .......... ..........

..........

_________________________________________________________________________

(m) C1 (Figure 16)

Discrete approximation to gamma distributed rates

Coefficient of variation of rates = 1.690000 (alpha = 0.350128)

States in HMM Rate of change Probability

1 0.143 0.667

2 1.746 0.256

3 5.148 0.067

4 10.489 0.0096

5 18.033 0.00066

6 28.254 0.000019

7 42.100 0.000000

8 62.030 0.000000

User-defined tree:

+---------Chlamydomonas

+----------------------7

| +---------------------Ostreococcus

|

+--3 +-------------Ginkgo

| | +--8

| | +---1 +-----Arabidopsis

| | | |

+------4 +---2 +---------------------------Pinus

| | |

| | +----Physcomitrella

| |

| | +-------Picea

| +------5

| +Vitis

|

6-Oryza

|

+-Zea

Ln Likelihood = -7460.55694

Between And Length Approx. Confidence Limits

------- --- ------ ------- ---------- ------

6 Zea 0.50142 ( 0.30842, 0.69440) **

6 4 1.37817 ( 0.68329, 2.07304) **

4 3 0.69413 ( 0.00013, 1.38814) **

3 7 4.66831 ( 2.99675, 6.33990) **

7 Chlamydomonas 2.03387 ( 0.81981, 3.24794) **

7 Ostreococcus 4.41088 ( 2.80798, 6.01376) **

3 2 0.85740 ( 0.07441, 1.64038) **

2 1 0.86112 ( 0.11931, 1.60292) **

1 8 0.46478 ( zero, 1.13110)

8 Ginkgo 2.89362 ( 1.86155, 3.92569) **

8 Arabidopsis 1.19748 ( 0.51124, 1.88370) **

1 Pinus 5.62010 ( 4.18341, 7.05678) **

2 Physcomitrella 1.10206 ( 0.44158, 1.76254) **

4 5 1.38231 ( 0.60705, 2.15756) **

5 Picea 1.70597 ( 1.11641, 2.29553) **

5 Vitis 0.22347 ( zero, 0.63318)

6 Oryza 0.19591 ( 0.03722, 0.35460) **

* = significantly positive, P < 0.05

** = significantly positive, P < 0.01

Combination of categories that contributes the most to the likelihood:

1111111111 1111111111 1111111121 1111111111 1111111111 1111111111

1111111111 1111111111 1111111111 1111111111 1111111211 1111211121

1111111111 1111211121 1111111111 1111111111 1111111211 1111111121

1111111111 1111111111 1111111111 1122221212 1121211211 2222221222

1212221222 1122222222 1111111111 1111111111 1111111111 1111111111

1112121121 1121111122 2112111211 1111112111 1222122212 2221121122

2212221121 2222112122 2122121122 2222122222 1222222122 2222222221

2211222222 1111212122 2212221212 2122212122 2221211122 2112111211

2122211121 1211122121 1122111121 1111211111 1111111111 2111111111

1111111111 1111111111 1111121111 1111111111 1111111111 1111111111

1111111111 1111111111 1111111111 1111111111 1111111111 1111111111

1111111111 1111111111 1111111111 1111111111 1111111111 11111

Most probable category at each site if > 0.95 probability ("." otherwise)

.......... ........11 11111.11.. .......... .......... ..........

.......... .......... .......... .......... ......1..1 1111..11..

1..111.111 1111.111.1 1111111111 1111111111 1111.11.11 11111111.1

1111111111 1111111111 11111111.1 1.....1.1. .1........ ......1...

..1...1... 1......... .......... .......... .......... ..........

..1....... .......... .......... .........1 .......... ..........

......1... .......... .......... .......... .......1.. .........1

...1...... .1........ .......... .....1.... ......11.. ..........

.....1.... ....1..... .......1.. .......... .......... ..........

.......... .......... .......... .......... .......... ..........

.......... .......... .......... .......... .......... ..........

.......... .......... .......... .......... .......... .....

_________________________________________________________________________

(n) AtMRP2 (Figure 17)

Discrete approximation to gamma distributed rates

Coefficient of variation of rates = 0.520000 (alpha = 3.698225)

States in HMM Rate of change Probability

1 0.250 0.084

2 0.615 0.368

3 1.133 0.386

4 1.823 0.142

5 2.713 0.020

6 3.854 0.00096

7 5.339 0.000013

8 7.415 0.000000

User-defined tree:

+--Oryza

|

+--3 +----Physcomitrella

| | |

| | | +----------------------------------Cyanothece

| +--2 +--6

| | | | +-----------Ostreococcus

| | +--5 +------7

| | | | +-----Chlamydomonas

| +---------1 |

| | +-------------------Bdellovibrio

| |

| +----------------Zea

|

4-Vitis

|

+-Arabidopsis

Ln Likelihood = -24101.18999

Between And Length Approx. Confidence Limits

------- --- ------ ------- ---------- ------

4 Arabidopsis 0.15642 ( 0.13079, 0.18202) **

4 3 0.04442 ( 0.02377, 0.06507) **

3 Oryza 0.18101 ( 0.15328, 0.20873) **

3 2 0.18960 ( 0.14774, 0.23147) **

2 Physcomitrella 0.30360 ( 0.25878, 0.34841) **

2 1 0.63799 ( 0.53653, 0.73945) **

1 5 0.12683 ( 0.04374, 0.20996) **

5 6 0.14558 ( 0.04556, 0.24542) **

6 Cyanothece 2.33053 ( 1.98238, 2.67865) **

6 7 0.43705 ( 0.32950, 0.54459) **

7 Ostreococcus 0.80780 ( 0.70870, 0.90687) **

7 Chlamydomonas 0.40232 ( 0.32463, 0.48001) **

5 Bdellovibrio 1.29337 ( 1.14619, 1.44053) **

1 Zea 1.14274 ( 1.01485, 1.27060) **

4 Vitis 0.08420 ( 0.06238, 0.10604) **

* = significantly positive, P < 0.05

** = significantly positive, P < 0.01

Combination of categories that contributes the most to the likelihood:

2233333333 3233323333 3333333333 2343433334 3244333333 3333333443

4333333333 3433343433 3443333333 3333332333 3233333323 3333343434

3333432233 3333333433 3333223333 4323433333 3333333333 3333334323

3333433333 3343432322 3443333333 3332333333 3334333433 2333333333

2333232234 3332323223 3334323333 3233423332 3233333333 4333333422

3333232322 2333333333 3222333223 2333221332 2322323343 2333333423

3333333333 3232233233 2332332233 2233323232 4332232322 2222222213

3322333323 2213222123 3222333422 3322223312 3233222432 2323332333

3323323234 2222343233 2223321312 3322232222 2221321213 2233323331

2313333233 3433233334 3323223223 3322332232 3322223221 2222232223

1233323222 3322232223 1233323321 3333333333 3333332232 2233222334

3433232233 3333333333 3333333333 3333333333 3333333332 3322332332

2222222322 3211123223 2222244223 3333333432 2321222132 2131222321

2222323333 3232223323 2431232223 2231212222 2121221111 1221112233

2222222212 1121222333 2233323332 2342132211 2222222332 3233233242

3332233323 3323423333 2333343344 3344423343 4334433343 3323333333

3344433343 3343423333 3333323332 3312123232 2331343234 2322333333

4332342333 3332333233 2332224323 3342333333 3433232234 2323333323

3223333432 3322332233 2242223222 2111221311 1221212132 3213322433

3323333333 2223232332 2333332232 2333334222 2233231222 2321232122

2232312231 2221122233 3222332343 2232223232 3332232233 2233223332

3332333333 3233233342 3223222323 1222223323 3322322331 2332221123

3233324122 3333322333 3322333222 3132333223 3212332131 1223224242

3311222121 1111113332 1213212331 2132122123 2322421123 2221212222

1312213122 2332232322 3222232323 3322334323 2223132312 1213111112

2221122222 2221211111 1112311221 1222233233 2122211112 2122131223

3233132324 3323322333 4233333232 3323223223 3333333333 3343333443

4433333223 2333333333 3333333332 2232323223 2232223333 3333332243

2423332334 3332323333 4333433333 4322323333 3322323232 3222233333

3333333342 3343333333 33

Most probable category at each site if > 0.95 probability ("." otherwise)

.......... .......... .......... .......... .......... ..........

.......... .......... .......... .......... .......... ..........

.......... .......... .......... .......... .......... ..........

.......... .......... .......... .......... .......... ..........

.......... .......... .......... .......... .......... ..........

.......... .......... .......... .......... .......... ..........

.......... .......... .......... .......... .......... ..........

.......... .......... .......... .......... .......... ..........

.......... .......... .......... .......... .......... ..........

.......... .......... .......... .......... .......... ..........

.......... .......... .......... .......... .......... ..........

.......... .......... .......... .......... .......... ..........

.......... .......... .......... .......... .......... ..........

.......... .......... .......... .......... .......... ..........

.......... .......... .......... .......... .......... ..........

.......... .......... .......... .......... .......... ..........

.......... .......... .......... .......... .......... ..........

.......... .......... .......... .......... .......... ..........

.......... .......... .......... .......... .......... ..........

.......... .......... .......... .......... .......... ..........

.......... .......... .......... .......... .......... ..........

.......... .......... .......... .......... .......... ..........

.......... .......... .......... .......... .......... ..........

.......... .......... .......... .......... .......... ..........

.......... .......... .......... .......... .......... ..........

.......... .......... .......... .......... .......... ..........

.......... .......... .......... .......... .......... ..........

.......... .......... .......... .......... .......... ..........

.......... .......... .......... .......... .......... ..........

.......... .......... ..

_________________________________________________________________________

(o) SEE2 (Figure 18)

Discrete approximation to gamma distributed rates

Coefficient of variation of rates = 0.780000 (alpha = 1.643655)

States in HMM Rate of change Probability

1 0.193 0.233

2 0.736 0.445

3 1.645 0.256

4 2.954 0.060

5 4.724 0.0059

6 7.061 0.00022

7 10.170 0.000002

8 14.588 0.000000

User-defined tree:

+Volvox

|

| +Oryza

| +--6

| | +Zea

| +--5

| | | +-Arabidopsis

| +--4 +--7

| | | +-Lycopersicon

| +----3 |

| | | +---Picea

1-----2 |

| | +---Physcomitrella

| |

| +---------------------------------------------------Ostreococcus

|

+-Chlamydomonas

Ln Likelihood = -6589.02623

Between And Length Approx. Confidence Limits

------- --- ------ ------- ---------- ------

1 Chlamydomonas 0.15937 ( 0.02721, 0.28676) **

1 Volvox 0.10928 ( zero, 0.24951) *

1 2 0.49327 ( 0.31759, 0.67635) **

2 3 0.39490 ( 0.22096, 0.56891) **

3 4 0.13626 ( 0.05825, 0.21416) **

4 5 0.13493 ( 0.07127, 0.19857) **

5 6 0.18977 ( 0.12910, 0.25044) **

6 Oryza 0.07329 ( 0.04031, 0.10627) **

6 Zea 0.09689 ( 0.06185, 0.13194) **

5 7 0.10223 ( 0.05141, 0.15306) **

7 Arabidopsis 0.20924 ( 0.15193, 0.26656) **

7 Lycopersicon 0.15919 ( 0.10779, 0.21060) **

4 Picea 0.32861 ( 0.24361, 0.41361) **

3 Physcomitrella 0.29086 ( 0.20234, 0.37938) **

2 Ostreococcus 4.34381 ( 3.28000, 5.40762) **

* = significantly positive, P < 0.05

** = significantly positive, P < 0.01

Combination of categories that contributes the most to the likelihood:

2222222222 2222222222 1332343323 2223233232 3323333323 2322222222

2222334433 3322223333 3332132221 2222222222 2222222223 2322433222

1121211132 2411112112 2111122222 2222222222 2222222222 2222222222

2112322122 1121122222 2222213232 1121131213 2313212312 2122111132

1223222222 2222312222 2222332222 2111223111 2122222221 1122111211

2323222222 2222222221 2221222212 2221322112 2132311111 1222222222

2222222222 2222222222 2222221221 2121322222 2112222222 2222222222

2222222222 2222222222 2222222222 2222222222 2222222222 2222222222

2222222222 2222222222 2222222222 2222222222 2211112111 2122222211

2222422222 2221112121 2122112223 2222222222 2222222222 2222222222

2321213212 3222321223 3123311111 2212343243 2222222222 2222222222

2222222222 2222222222 2222232333 3132222222 2222222232 3233332222

4333333222 2111122332 2323232333 1322322222 2222222222 2222222123

3232322213 2213222222 2222222222 2222222222 2222222222 2222222222

2222222222 2222422233 2222342222 2222213312 2222222222 2222222222

2222211223 2222312222 3222222222 2112222211 2221212212 2111223223

2222333223 2222322332 1233322112 2222222222 2222222222 2222222222

2222222222 222

Most probable category at each site if > 0.95 probability ("." otherwise)

.......... .......... .......... .......... .......... ..........

.......... .......... .......... .......... .......... ..........

.......... ..1..1.... .......... .......... .......... ..........

.......... .......... .......... 1......... .......... ..........

.......... .......... .......... .......... .......... ..........

.......... .......... .......... .......... .......... ..........

.......... .......... .......... .......... .......... ..........

.......... .......... .......... .......... .......... ..........

.......... .......... .......... .......... .......... ..........

.......... .......... .......... .......... .......... ..........

.......... .......... .......... .......... .......... ..........

.......... .......... .......... .......... .......... ..........

.......... .......... .......... .......... .......... ..........

.......... .......... .......... .......... .......... ..........

.......... .......... .......... .......... .......... ..........

.......... .......... .......... .......... .......... ..........

.......... .......... .......... .......... .......... ..........

.......... ...
