# Supplementary material for: Evolution of plant senescence
Source: BMC Evol Biol. 2009 Jul 14;9:163. doi: 10.1186/1471-2148-9-163 (PMC2716323; doi:10.1186/1471-2148-9-163)
Supplement: Additional file 3 — Synopsis of significant events in the evolution of plant senescence in relation to the geological timeline. Innovations in the geological time-line relevant to the evolution of a senescence program integrated into plant development and adaptation. [file 1471-2148-9-163-S3.doc]

**Additional File 1**. Synopsis of significant events in the evolution of plant senescence in relation to the geological timeline.

| **Period** | **Dominant stress** | **Stage of evolution** | **Key event or process** | **Legacy** |
| --- | --- | --- | --- | --- |
| Archean - Paleo-proterozoic eons  3800-1500 mya | Competition for light | Pre-endosymbiotic | Light-driven charge separation | Proton pump  ATP generation  Quenching (carotenoids, assimilation, eventually water-splitting) |
|  |  |  | Assembly of pigment-protein complexes | Turnover  Proteolysis  Chaperones, facilitated shape-matching |
|  |  |  | Mixotrophy | Disposal of photosynthetic apparatus  Nutrient transporters |
|  |  |  | Horizontal gene transfer | Near universal distribution of genes for the above  Intractable phylogenies |
| Mesoproterozoic - Ordovician  1500-450 mya | Competition for nutrients | Endosymbiotic | Mixotrophy | Plastid retains capacity for auto- or hetero-trophy  Modulation of turnover between assembly and disassembly of complexes |
|  |  |  | Autolysis | Intracellular sequestration of ‘disasterases’  Self-destruction  Origins of PCD |
|  |  |  | Excretion | Secretion of antibiotics and allelopathic chemicals  Elimination of phototoxic chlorophyll products |
|  |  |  | Sex | Beginnings of cell specialisation  Production of resting bodies  Beginnings of nutrient salvage and stress avoidance  Beginnings of tractable phylogenies |
| Ordovician - Silurian  450-410 mya | Immobility | Multicellular | Polarity | Awareness of self in relation to environment  Anisotropic growth and development |
|  |  |  | Cell specialisation | Beginnings of sources and sinks  Limitations on cell versatility and autonomy  Morphologies generated by hydraulics and rigid cell walls  First appearance of tissues |
|  |  |  | Transport systems | Movement of materials around plant body  Development of specialised transport tissues |
| Silurian - Jurassic  410-150 mya | Unbalanced light capture and utilisation capacity | Early terrestrial | Life-support structures | Epidermis covers body with layer simulating ancestral aquatic environment  Vacuole a souvenir of ancestral environment inside every cell |
|  |  |  | Enhanced requirement for light energy dissipation | Xanthophyll cycle  Promiscuous CO_2_ fixation  Resource rejection |
|  |  |  | Nutrient salvage | Tissue differentiation in sources and sinks  Elaboration of transport systems  Intracellular detoxification mechanisms, including for chlorophyll catabolites  Involvement of vacuole |
|  |  |  | Mixotrophic development | Plastid transdifferentiation network  Switch between light- and respiration-dependent energy supply |
|  |  |  | Elaboration of morphology and anatomy | Specialisation of organs  Exploitation of lysigeny and schizogeny in morphogenesis  Variations on common organ development theme  Organ shedding and beginnings of throw-away lifestyle |
| Jurassic - Anthropocene  150-0 mya | Challenges from other organisms | Tracheophytic | Adaptations to resist abiotic stresses | Mechanisms to resist excess light (sunblockers, quenchers)  Repair mechanisms |
|  |  |  | Adaptations to avoid abiotic stresses | Proliferation of life-forms  Resting and storage structures |
|  |  |  | Adaptations to competing or hostile organisms | Neighbour detection  Co-evolved signalling mechanisms  Visual, chemical and other cues  Mimicry  Disposal of parts |
|  |  |  | Adaptations to beneficial organisms | Attraction of pollinators, dispersers, defenders  Visual and chemical signals  Rewards |
|  |  |  | Human intervention | Domestication traits  Weed traits  Hypertrophy and low competitiveness  Exaggerated colours and shapes  Extreme introgressive and engineered gene transfer  Climate and environmental change  Extinctions and loss of biodiversity |
