# Supplementary material for: Evolution of plant senescence
Source: BMC Evol Biol. 2009 Jul 14;9:163. doi: 10.1186/1471-2148-9-163 (PMC2716323; doi:10.1186/1471-2148-9-163)
Supplement: Additional file 4 — Log Likelihood (Ln) values corresponding to Hidden Markov Model, Gamma Distribution Model and Constant Rate Variation options in PHYLIP for phylogenetic analysis of senescence-related protein sequences. Results of analysing all amino acid sequence data with the PROML program run with three different settings for the R (rate variation among sites) parameter. [file 1471-2148-9-163-S4.doc]

**Additional File 2.** Log Likelihood (Ln) values corresponding to Hidden Markov Model, Gamma Distribution Model and Constant Rate Variation options in PHYLIP for phylogenetic analysis of senescence-related protein sequences. Details of the software and approaches used to generate these data are given in the **Methods** section.

| Reference amino acid sequence | User-defined HMM  of rate (Ln) | Gamma distribution  (Ln) | Constant rate of  change (Ln) |
| --- | --- | --- | --- |
|
| RCCR  (NP_195417) | -5232.64 | -5231.84 | -5288.86 |
| PaO  (NP_190074) | -10607.61 | -10587.44 | -10801.95 |
| Sgr1  (NP_567673) | -5433.39 | -5395.48 | -5587.53 |
| WBC23  (NP_850781) | -10702.75 | -10680.22 | -10920.53 |
| Wrky53  (NP_194112) | -5598.25 | -5536.51 | -5624.34 |
| AtNAP  (NP_564966) | -3011.78 | -2992.59 | -3087.44 |
| dee4  (Q9XFY8) | -5824.19 | -5760.42 | -5904.97 |
| Fibrillin  (NP_192311) | -6348.39 | -6315.54 | -6428.72 |
| CCD8  (NP_195007) | -15481.69 | -15459.79 | -15678.18 |
| OrI  (ABH07405) | -5438.32 | -5447.81 | -5519.64 |
| OrII  (NP_851031) | -5382.33 | -5396.81 | -5476.99 |
| Bronze1  (P16167) | -6963.68 | -6911.81 | -7020.46 |
| myb C1  (P10290) | -8248.98 | -8238.37 | -8434.98 |
| AtMRP2  (NP_181013) | -24026.79 | -23900.66 | -24403.61 |
| See2  (CAB64545) | -8629.78 | -8547.96 | -8708.51 |
